# Identification of Leg Chemosensory Genes and Sensilla in the *Apolygus lucorum*

**DOI:** 10.3389/fphys.2020.00276

**Published:** 2020-04-15

**Authors:** Zibo Li, Yaoyao Zhang, Xingkui An, Qi Wang, Adel Khashaveh, Shaohua Gu, Shun Liu, Yongjun Zhang

**Affiliations:** ^1^State Key Laboratory for Biology of Plant Diseases and Insect Pests, Institute of Plant Protection, Chinese Academy of Agricultural Sciences, Beijing, China; ^2^College of Plant Protection, Agricultural University of Hebei, Baoding, China; ^3^College of Plant Protection, China Agricultural University, Beijing, China

**Keywords:** *Apolygus lucorum*, legs, transcriptome sequencing, chemosensory genes, expression profiles, sensilla

## Abstract

*Apolygus lucorum* (Hemiptera: Miridae), one of the main insect pests, causes severe damage in cotton and many other economic crops. As is well-known, legs play important roles in the chemoreception of insects. In this study, the putative chemosensory proteins in legs of *A. lucorum* involved in close or contact chemical communication of adult bugs were investigated using RNA transcriptome sequencing and qPCR methods. Transcriptome data of forelegs, middle legs and hind legs of adult bugs demonstrated that 20 odorant binding protein (OBP) genes, eight chemosensory protein (CSP) genes, one odorant receptor (OR) gene, one ionotropic receptor (IR) gene and one sensory neuron membrane protein (SNMP) gene were identified in legs of *A. lucorum*. Compared to the previous antennae transcriptome data, five CSPs, IR21a and SNMP2a were newly identified in legs. Results of qPCR analysis indicated that all these putative chemosensory genes were ubiquitously expressed in forelegs, middle legs and hind legs of bugs. Furthermore, four types of sensilla on legs of *A. lucorum* including sensilla trichodea (subtypes: long straight sensilla trichodea, Str1; long curved sensilla trichodea, Str2), sensilla chaetica (subtypes: sensilla chaetica 1, Sch1; sensilla chaetica 2, Sch2; and sensilla chaetica 3, Sch3), sensilla basiconca (subtypes: medium-long sensilla basiconca, Sba1; short sensilla basiconca, Sba2) and Böhm bristles (BB) were found using scanning electron microscopy. Additionally, the largest number of sensilla was observed on hind legs, while the forelegs had the smallest number of sensilla. Our data provide valuable insights into understanding the chemoreception of legs in *A. lucorum.*

## Introduction

Insects rely on their sensitive olfactory and gustatory organs to find hosts, forage, lay eggs and mate (Romani et al., 2005; Li and Liberles, 2015). Lots of chemosensory sensilla are independently distributed in various organs of insects such as antennae, mouthparts, legs, wings and ovipositors. Insect legs sense chemical signals when the insects land on the host (Klijnstra and Roessingh, 1986; Maher et al., 2006). Legs of *Drosophila* are involved in making the preliminary contact with food resources and non-volatile pheromones (Frederick et al., 2014; Koh et al., 2014). Likewise, legs of *Helicoverpa armigera* can sense some salts, sugars and amino acids (Zhang et al., 2010). Generally, chemosensory associated proteins in insect antennae such as odorant binding proteins (OBPs), chemosensory proteins (CSPs), Niemann-Pick type C2 proteins (NPC2s), odorant receptors (ORs), gustatory receptors (GRs), ionotropic receptors (IRs) and sensory neuron membrane proteins (SNMPs) play crucial roles in olfactory and gustatory behaviors of insects (Pelosi et al., 2006; Wynand and Carlson, 2006; Abdel-Latief, 2007; Wanner and Robertson, 2008; Benton et al., 2009; Touhara and Vosshall, 2009; Vogt et al., 2009; Leal, 2013; Adachi et al., 2014). These chemosensory associated proteins are usually also expressed in insect legs. By transcriptome sequencing, OBPs, CSPs, SNMPs, GRs, and ORs were identified in the legs of *Apis cerana cerana, Ectropis obliqua*, and *Adelphocoris lineolatus* (Ma et al., 2016; Sun et al., 2017; Du et al., 2019).

OBPs as carrier proteins play an indispensable role in transport of chemical signals through the aqueous sensilla lymph to the olfactory receptor cells in insects (Calvello et al., 2003; Pelosi et al., 2006). OBPs can recognize and distinguish different odorant messages (Schultze et al., 2012). LUSH, one of the *Drosophila* OBPs, is required for activation of pheromone-sensitive chemosensory neurons (Xu et al., 2005). Additionally, OBPs are essential for mediating olfactory behavioral responses. When the expression of OBPs was suppressed in flies, their behavioral responses to odorants were changed (Swarup et al., 2011). CSPs highly expressed in the sensilla lymph exhibit binding activities to odorants and pheromones, suggesting their olfactory functions (Pelosi et al., 2006; Hua et al., 2013; Sun et al., 2015).

Insect ORs play crucial roles in detecting the volatile molecules, especially in the long distance perception (Missbach et al., 2014). Generally, a typical OR unit functions as a dimer complex with the highly conserved odorant receptor co-receptor (Orco) among insect species (Wicher et al., 2008). In insects, GRs expressed in gustatory neurons acts as taste receptors, which are responsible for feeding behaviors (Dunipace et al., 2001; Scott et al., 2001). As a large and highly divergent family of ionotropic glutamate receptors in *Drosophila*, IRs are subdivided into “antennal IRs,” which are expressed in antennae specifically and mainly involved in olfactory recognition; and “divergent IRs,” which are found in various tissues taking responsible for sensation of taste (Jaeger et al., 2018; Lee et al., 2018; He et al., 2019; Rimal et al., 2019). Various chemosensory associated proteins in insect play diverse functions, such as those listed in Table 1.

**TABLE 1:** Diverse functions of chemosensory associated proteins in insects reported in previous studies.

Chemosensory associated proteins	Insect species	Function	References
LUSH	*Drosophila*	Olfaction	Xu et al., 2005
AlinOBP11	*Adelphocoris lineolatus*	Gustation	Sun et al., 2016
NPC2a	*Microplitis mediator*	Olfaction	Zheng et al., 2017
OR28	*Apolygus lucorum*	Olfaction	Yan et al., 2015
GR64a	*Drosophila melanogaster*	Gustation	Dahanukar et al., 2007
GR66	*Bombyx mori*	Gustation	Zhang et al., 2019
SNMP1	*Bombyx mori*	Olfaction	Zhang et al., 2018
IR94b	*Drosophila*	Audition	Senthilan et al., 2012
IR20a	*Drosophila*	Olfaction	Koh et al., 2014

There are different types of chemosensory sensilla distributed on insect legs. Gustatory sensilla such as contact sensilla chaetica on tarsi enable insects to perceive taste substances on host plant surfaces (Ave et al., 1978). Enormous amount of sensilla were observed on the antennae of *Lygus lineolaris* and *Apolygus lucorum* (Chinta et al., 1997; Lu et al., 2007). However, little is known about the sensilla types of legs of mirid bugs so far.

The *A. lucorum*, one of the dominant mirid bug species, has become a major pest in cotton fields along with the widespread cultivation of Bt transgenic cotton, resulting in substantial economic losses (Lu et al., 2010). Moreover, it is very difficult to control *A. lucorum* due to their polyphagous host-feeding and host transfer behaviors (Lu and Wu, 2008). In the present study, transcriptome sequencings of forelegs, middle legs and hind legs form adult *A. lucorum* were performed to identify the candidate chemosensory genes. The tissue- and sex-biased expression patterns of the putative chemosensory genes were assessed by conducting quantitative real-time PCR (qPCR). Moreover, the sensilla types on legs of adult male and female bugs were observed and characterized. Our finds provide valuable insights into understandings the roles of mirid bug legs in olfaction and gustation.

## Materials and Methods

### Insects Rearing and Tissue Collection

Nymphs and adults of *A. lucorum* were collected from cotton fields at the Langfang Experimental Station of Chinese Academy of Agricultural Sciences, Hebei Province (39.53° N, 116.70° E), China. Laboratory colonies feeding on green beans (*Phaseolus vulgaris* L.) were bred in climate chambers under following conditions: 29 ± 1°C, relative humidity (RH) 60 ± 5% and 14: 10 light: dark (L: D) photoperiod (An et al., 2016).

For transcriptome sequencing, 500 forelegs, 500 middle legs and 500 hind legs were collected from 5 days old adult bugs of both sexes. In qPCR measurement, forelegs, middle legs and hind legs samples from *A. lucorum* of both sexes were separately collected and immediately immersed in liquid nitrogen till to use.

### cDNA Library Construction and Transcriptome Sequencing

Total RNAs were isolated from samples using Trizol regent (Invitrogen, Carlsbad, CA, United States) following the manufacturer’s instructions. RNA purity was checked using NanoDrop^®^ spectrophotometers (Thermo Fisher, Waltham, MA, United States). RNA integrity was assessed using the RNA Nano 6000 Assay Kit (Agilent Technologies, Santa Clara, CA, United States). The cDNA library construction and transcriptome sequencing were carried out by Sinobiocore Bioinformatics Technology Co. Ltd on an Illumina HiSeq 2500 platform (Beijing, China).

A total amount of 1 μg RNA per sample was used as input material for the library preparation. The sequencing libraries were generated using the TruSeq RNA Sample Preparation Kit (Illumina, San Diego, CA, United States). Briefly, mRNA was purified from total RNA using poly-T oligo-attached magnetic beads. Fragmentation was performed using divalent cations under elevated temperature in an Illumina proprietary fragmentation buffer. First strand cDNA was synthesized using random oligonucleotides and SuperScript II (Thermo Fisher, Waltham, MA, United States). Second strand cDNA synthesis was performed using DNA Polymerase I and RNase H. Then, the cDNA fragments were end repaired with the addition of a single ‘A’ base at the 3′-end of each strand, ligated with the special sequencing adapters subsequently. The products were purified and size selected in order to get appropriate size for sequencing. Finally, PCRs were performed and aimed products were purified.

Library concentration was measured using Qubitp^®^ RNA Assay Kit in Qubitp^®^ 3.0 (Thermo Fisher, Waltham, MA, United States) to preliminary quantify. Insert size was assessed using the Agilent Bioanalyzer 2100 system (Agilent Technologies, Santa Clara, CA, United States). When the insert size was in consistent with expectations, qualified insert size was accurately quantified using qPCR by Step One Plus realtime PCR system (Applied Biosystems, Foster City, CA, United States). The clustering of the index-coded samples was performed on a cBot Cluster Generation System (Illumia, San Diego, CA, United States) according to the manufacturer’s instructions. After cluster generation, the library preparations were sequenced on an Illumina Hiseq 2500 platform with 150 bp paired-end module.

### Transcriptome Assembly and Functional Annotation

*De novo* transcriptome assembly was performed using Trinity^1^. A K-mer library was constructed with the filtered reads, and the contigs were formed using Inchworm. Cuffdiff (v2.2.1) was used to calculate FPKMs for coding genes in each sample. Gene FPKMs were computed by summing the FPKMs of the transcripts in each gene group. FPKM stands for “fragments per kilobase of exon per million fragments mapped,” and it is calculated based on the length of the fragments and the reads count mapped to each fragment (Anders et al., 2015). To annotate the unigenes, blastx and blastn searches were performed against the database of Nt, Nr, SwissProt, and eggNOG (*e*-value < 10^–5^, bitscore > 60)^2^. The blast results were then imported into Blast2GO pipeline for Go annotations.

### Differential Expression Analysis

Deseq2 provides statistical routines for determining differential expression in digital transcript or gene expression datasets using a model based on a negative binomial distribution. Genes with corrected *p*-values less than 0.05 and the absolute value of log2 (fold change) < 1.0 were assigned as significantly differentially expressed (Love et al., 2014). Differentially expressed genes (DEGs) were identified by Benjamini and Hochberg FDR according to statistically significant differences with the threshold of false discovery rates (FDR) < 0.05 and Fold Change ≥ 2 (Leng et al., 2013; Nikolayeva and Robinson, 2014).

### Identification of Putative Chemosensory Genes

In addition to keywords searching, a FASTA file of unigenes was created from a local nucleotide database file using the BioEdit Sequence Alignment Editor program 7.13, and the local BLASTN program was performed using available *A. lucorum* antennae chemosensory genes (unpublished data) as the queries. Candidate unigenes encoding putative chemosensory genes were used BLASTX to search in NCBI website (Zhou et al., 2010).

### Verification of Candidate Genes

All the sequences of candidate chemosensory genes from transcriptome were further confirmed by gene cloning and sequencing. Gene-specific primers (Supplementary Table S1) amplifying the full length or partial sequences of target genes were designed using Primer Premier 5.0 (PREMIER Biosoft International, Palo Alto, CA, United States). PCR reactions were carried out in a volume of 50 μl with 200 ng cDNA template of each sample and 1 μl TransStartp^®^ FastPfu DNA Polymerase (TransGen Biotech, Beijing, China). The PCR parameters were: 95°C for 1 min, followed by 40 cycles of 95°C for 20 s, 55°C for 20 s, 72°C for 1 min, and a final extension at 72°C for 5 min. PCR products were subsequently gel-purified and cloned into pEASY^®^-Blunt Cloning vector (TransGen Biotech, Beijing, China) and then sequenced with standard M13 primers.

### Phylogenetic Analysis of CSPs

Multiple alignments of the complete CSPs amino acid sequences were performed by ClustalX 2.0 and further edited by GeneDoc 2.7. The phylogenetic tree was constructed by MEGA 7.0 using the Neighbor-joining method with a p-distance model and a pairwise deletion of gaps. Bootstrap support was assessed by a bootstrap procedure based on 1000 replicates (Felsenstein, 1985; Saitou and Nei, 1987; Nei and Kumar, 2000; Kumar et al., 2016). The data sets of CSPs sequences which were chosen from other hemipteran species (Supplementary Table S2).

### The qPCR Analysis

The relative expression levels of candidate chemosensory genes in forelegs, middle legs and hind legs of both sexes were examined by qPCR on an ABI Prism 7500 system (Applied Biosystems, Carlsbad, CA, United States). Reaction system contained a mixture of 10 μl 2 × SuperReal PreMix Plus (Tiangen Biotech, Beijing Co., Ltd.), 0.6 μl of each primer (10 μM), 200 ng sample cDNA, 0.4 μl 50 × ROX Reference Dye and proper volume of RNase-free water. PCR cycling parameters were as follows: 95°C for 15 min, followed by 40 cycles of 95°C for 10 s, cooled to 60°C for 32 s. Then, the PCR products were heated to 95°C for 15 s, cooled to 60°C for 1 min, heated to 95°C for 30 s and cooled to 60°C for 15 s to measure the dissociation curves. The *GAPDH* (GenBank accession No. JX987672) of *A. lucorum* stably expressed in different tissues (Supplementary Figure S1) was used as a reference gene for normalization (Ji et al., 2013). Primers (Supplementary Table S3) of the target and reference genes were designed using the Beacon Designer 7.9 (PREMIER Biosoft International, Palo Alto, CA, United States). A discrete amplification peak and a subsequent melting curve were evaluated to ensure the primer specificity. Each qPCR reaction for each sample was performed in three technical replicates and three biological replicates. Five serial tenfold dilutions of cDNA from each sample were amplified. For each dilution, amplifications were performed in triplicate using primers for the target gene and *GAPDH*. As a result, the absolute values of the slopes of all lines from template dilution plots (log cDNA dilution vs. ΔCT) were close to zero, indicating that the amplification efficiencies of the target and reference genes were approximately equal. Then, the comparative 2^–Δ^ ^Δ^ ^*CT*^ method (Livak and Schmittgen, 2001) was used to calculate the relative expressions of different tissue samples. The comparative analyses of each target gene among various samples and sexes were determined using a two-way analysis of variance (ANOVA), followed by least significant difference (LSD) post-hoc test, *P* < 0.05 using the IBM SPSS Statistics 25.0 software (SPSS Inc., Chicago, IL, United States).

### Observation of Sensilla on Legs of *A. lucorum*

The sensilla types on legs of *A. lucorum* were observed using a scanning electron microscopy (GeminiSEM 500, Zeiss, Germany). Forelegs, middle legs and hind legs were removed from female and male adult *A. lucorum*, respectively. Leg samples were fixed in 70% ethanol for 3 h, cleaned in an ultrasonic bath (250 W) for 10 s and finally subjected to gradient elution in an ethanol series (70, 80, 90, 95, and 100%). Subsequently, samples were dried in an oven thermostat at 25°C for 10 h. After coated with gold-palladium and mounting on holders, samples were observed under a scanning electron microscopy. Identification of the leg sensilla was mainly based on the description of Chinta et al. (1997) and Lu et al. (2007).

## Results

### Overview of Transcriptome

**FIGURE 1:**
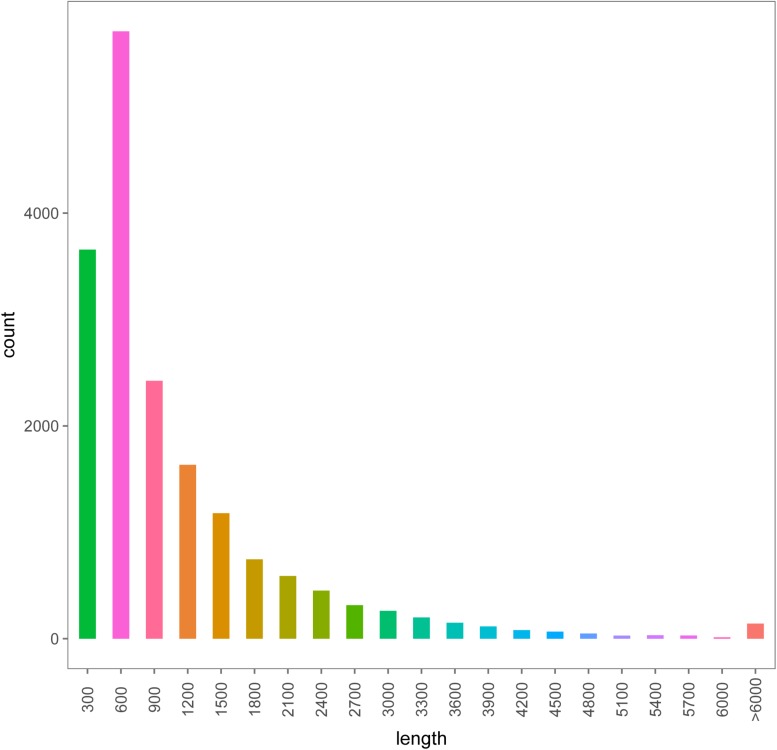
The length distributions of the assembled unigenes from legs transcriptomes of female and male *A. lucorum*.

Six transcriptome data from forelegs, middle legs and hind legs of male and female were generated by HiSeq 2500 platform. A total of 106,020,038, 96,631,626, 110,435,520, 104,039,236, 90,768,110, and 106,745,344 raw reads were produced from six female and male leg samples (forelegs, middle legs, and hind legs), respectively. After filtering the low quality and adaptor sequences, we obtained 102,528,364, 93,578,672, 106,681,660, 100,468,622, 87,850,168, and 103,088,688 clean reads, respectively (Supplementary Tables S4, S5). The assembly of all clean reads together led to the generation of 40,968,256 contigs with a mean length of 1,071 bp. After merging and clustering, 17,459,594 unigenes with a mean length of 977 bp and N50 of 1,587 bp were acquired (Table 2). Of the clean reads, the Q30 percentage (proportion of sequences with a sequencing error rate less than 0.1%) for both libraries exceeded 94% (Supplementary Tables S4, S5). The length distributions of the unigenes were listed in Figure 1.

**TABLE 2:** Summary of total legs transcriptomes assembly.

Statistics project	Trinity contig	Unigenes
Minimum length	200 bp	200 bp
Mean length	1,071 bp	977 bp
Median length	669 bp	562 bp
Max length	21,715 bp	21,715 bp
N50	1,701 bp	1,587 bp
N90	440 bp	383 bp
Total assembled bases	40,968,256	17,459,594

**FIGURE 2:**
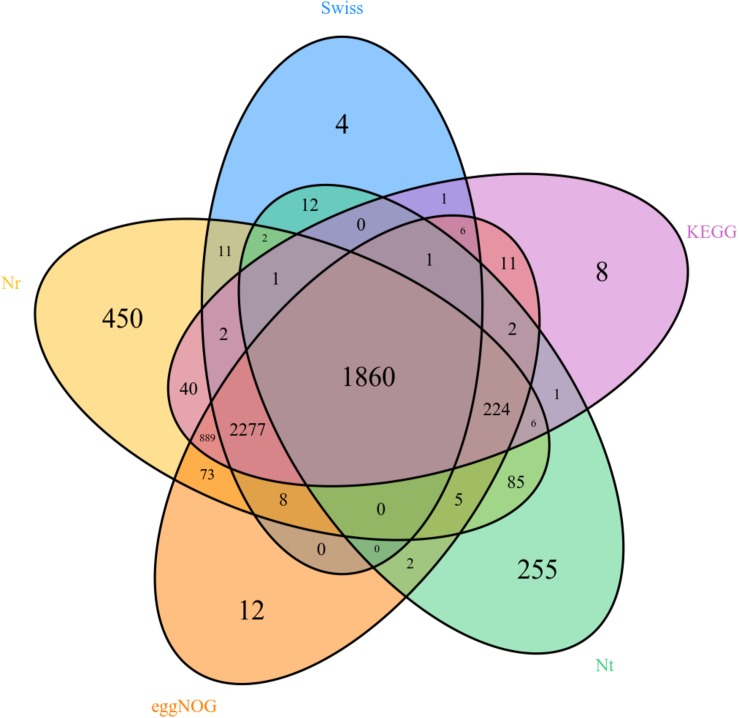
Venn diagram of comparison results of Nr, Nt, eggNOG, and Swissprot.

Unigenes were searched with blastx and blastn programs against the sequences in the NCBI GenBank database. The results showed that 5,933 out of the 17,459,594 unigenes had blastx hits in the non-redundant protein (nr) databases, and 2,456 unigenes had blastn hits in the non-redundant nucleotide sequence (nt) databases. Some unigenes were homologous to more than one species, and most of the annotated unigenes had the best hit with hemipteran insect genes (Figure 2). There are 358, 418, 562, 669, 1,347, 1,812, 2,186, 3,141, and 3,519 different genes in female forelegs vs. female middle legs, male middle legs vs. female middle legs, male middle legs vs. male forelegs, female forelegs vs. male forelegs, female hind legs vs. female middle legs, female hind legs vs. female forelegs, female hind legs vs. male hind legs, male middle legs vs. male hind legs, and male hind legs vs. male forelegs, respectively (Supplementary Table S6).

**FIGURE 3:**
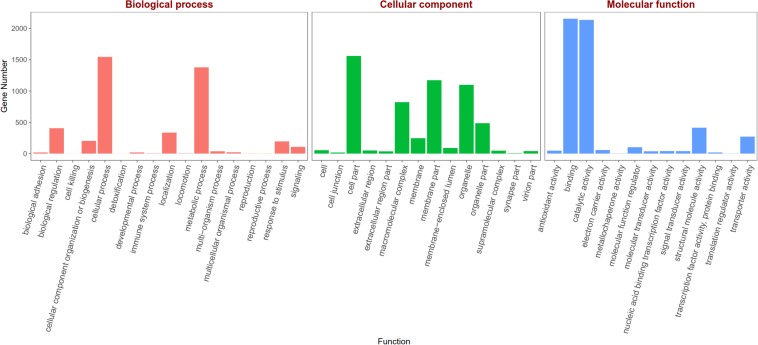
Gene Ontology (GO) classifications of legs transcripts annotated at GO level 2 according to the involvement in biological processes, cellular component and molecular function.

**FIGURE 4:**
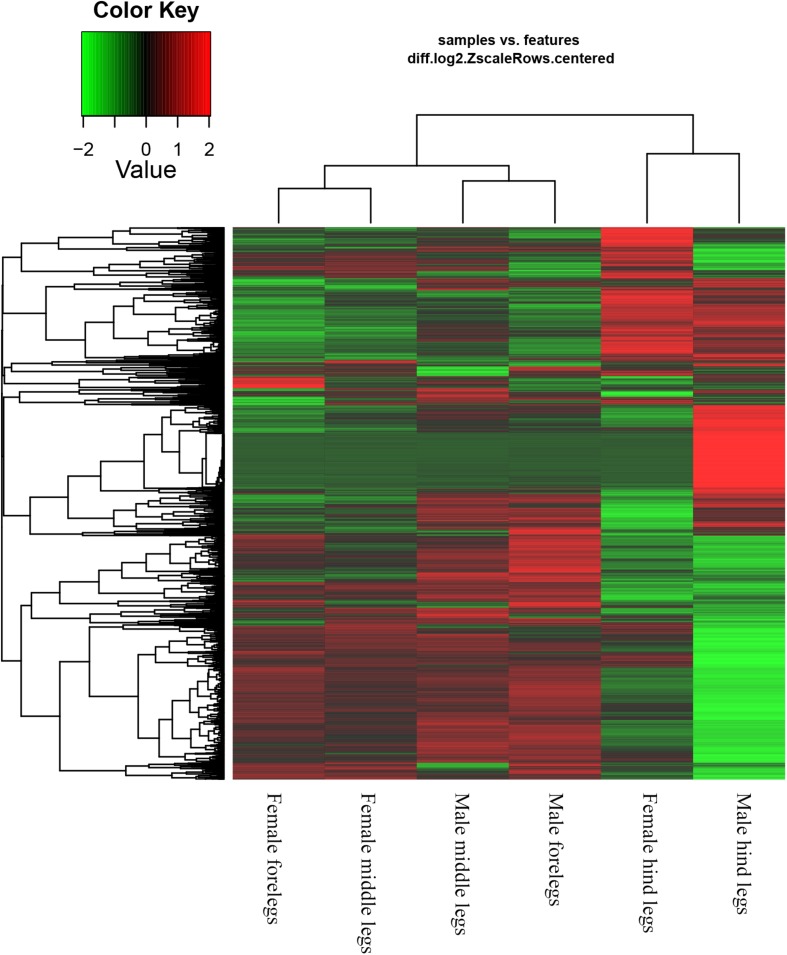
Hierarchical clustering results of differentially expressed genes between sample groups.

Based on the Gene Ontology (GO) annotations, 4,844 unigenes could be annotated into the following three functional categories: molecular function, cellular components and biological processes. The cellular process (31.92% unigenes) and metabolic process (28.47% unigenes) GO categories were most abundantly represented within the biological process GO. In the cellular components GO, the transcripts were mainly distributed in the membrane part (24.22% unigenes) and cell part (32.23% unigenes). The GO analysis also showed that the unigenes involved in binding (44.51% unigenes) and catalytic activity (44.12% unigenes) were most abundant in the molecular function ontology (Figure 3). The difference clustering results among six samples showed that there was a high correlation between female forelegs and female middle legs, male forelegs and male middle legs, female hind legs and male hind legs, separately (Figure 4).

### Putative Chemosensory Genes in *A. lucorum* Legs

**FIGURE 5:**
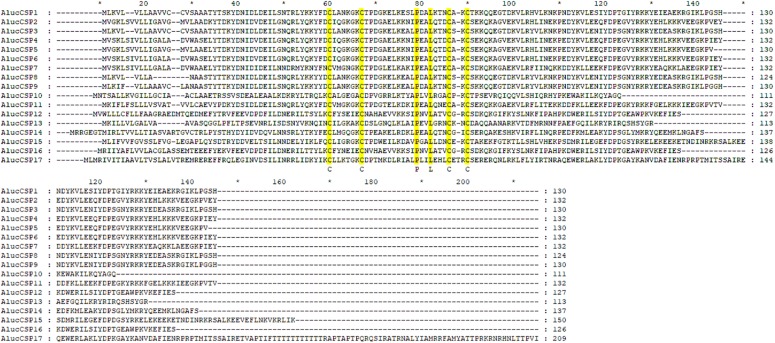
Multiple sequences alignment of CSPs of *A. lucorum*. Amino acid sequences were aligned by ClustalX 2.1 and edited by GeneDoc 2.7.0 software. Yellow boxes show conserved cysteine residues and other conserved residues. Accession numbers of CSPs of *A. lucorum* are listed in Table 2.

**FIGURE 6:**
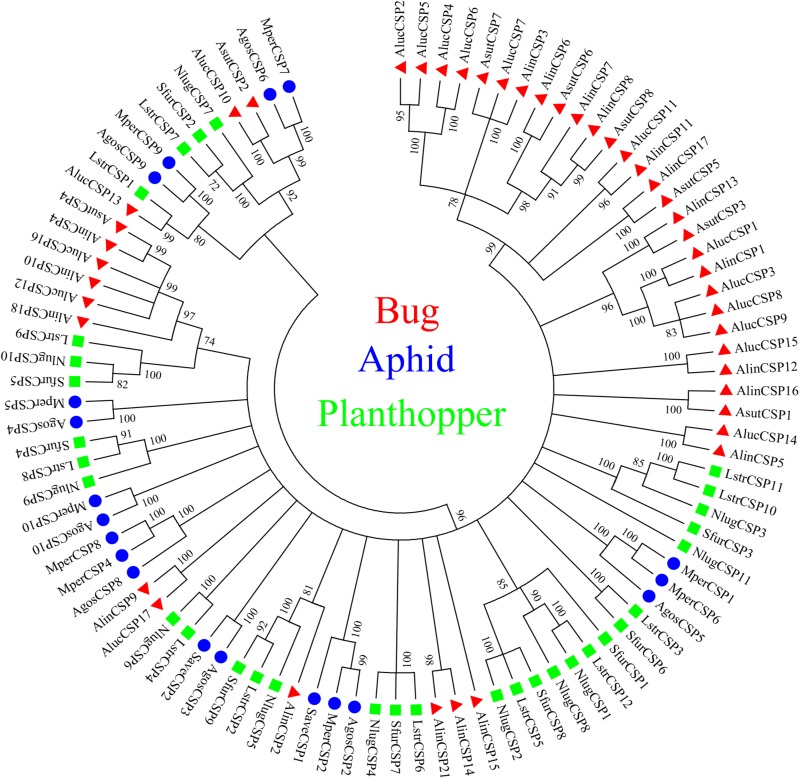
Phylogenetic analysis of CSPs. The CSP sequences used in this analysis are listed in Supplementary Table S4. Species abbreviations: Alin, *Adelphocoris lineolatus*; Aluc, *Apolygus lucorum*; Asut, *Adelphocoris suturalis*; Agos, *Aphis gossypii*; Mper, *Myzus persicae*; Save, *Sitobion avenae*; Sfur, *Sogatella furcifera*; Nlug, *Nilaparvata lugens*; Lstr, *Laodelphax striatellus*.

A total of 20 OBP genes, eight CSP genes, one OR gene, one IR gene, and one SNMP gene were identified (Table 3) in cDNA library of bug legs. Among these candidate chemosensory genes, *Aluc-OBP*2, 3, 4, 7, 8, 9, 11, 15, 16, 17, 18, 19, 22, 23, 26, 27, 28, 29, 31, 35; *CSP*2, 3, 4 and *OR*109 were previously reported in antennal transcriptome of *A. lucorum* (Hua et al., 2013; Yuan et al., 2015; An et al., 2016). However, *CSP*9, 10, 12, 16, 17, *IR*21a and *SNMP*2a (Table 3) were newly found and deposited in GenBank (accession numbers: MH781728, MH781729, MH781731, MN395398, MN395399, MH781745, and MH781750, respectively). All five candidate AlucCSPs represented the typical character of insect CSPs (Figure 5). In phylogenetic tree of 95 CSP sequences from hemipteran species (Supplementary Table S2), most of CSPs from same family were located in the same branch, whereas AlucCSP10, AlucCSP12, AlucCSP13, AlucCSP16, and AlucCSP17 were segregated into different central clusters (Figure 6).

**TABLE 3:** Chemosensory genes in male- and female- legs of *A*. *lucorum*.

Genes	Accession number	Best blastx hit				
		Putative genes	Species	Protein ID	*E*-value	Identity (%)
OBP2	HQ631398	Odorant binding protein 2	*Apolygus lucorum*	AEA07706.1	9E-111	100%
OBP3	HQ631399	Odorant binding protein 3	*Apolygus lucorum*	AEA07661.1	1E-71	92%
OBP4	HQ631400	Odorant binding protein 4	*Apolygus lucorum*	AEA07662.1	2E-108	98%
OBP7	JQ675724	Odorant binding protein 7	*Apolygus lucorum*	AFJ54048.1	7E-81	99%
OBP8	JQ675725	Odorant binding protein 8	*Apolygus lucorum*	AFJ54049.1	4E-95	96%
OBP9	JQ675726	Odorant binding protein 5	*Apolygus lucorum*	AEP95759.1	5E-102	99%
OBP11	JQ675728	Odorant binding protein 11	*Apolygus lucorum*	AFJ54052.1	9E-70	73%
OBP15	KT281923	Odorant binding protein 15	*Apolygus lucorum*	AMQ76468.1	4E-87	99%
OBP16	KT281924	Odorant binding protein 16	*Apolygus lucorum*	AMQ76469.1	4E-90	97%
OBP17	KT281925	Odorant binding protein 17	*Apolygus lucorum*	AMQ76470.1	6E-88	98%
OBP18	KT281926	Odorant binding protein 18	*Apolygus lucorum*	AMQ76471.1	3E-98	100%
OBP19	KT281927	Odorant binding protein 19	*Apolygus lucorum*	AMQ76472.1	4E-90	99%
OBP22	KT281930	Odorant binding protein 22	*Apolygus lucorum*	AMQ76475.1	5E-97	100%
OBP23	KT281931	Odorant binding protein 23	*Apolygus lucorum*	AMQ76476.1	6E-80	99%
OBP26	KT281934	Odorant binding protein 26	*Apolygus lucorum*	AMQ76479.1	1E-60	94%
OBP27	KT281935	Odorant binding protein 27	*Apolygus lucorum*	AMQ76480.1	3E-49	95%
OBP28	KT281936	Odorant binding protein 28	*Apolygus lucorum*	AMQ76481.1	6E-88	100%
OBP29	KT281937	Odorant binding protein 29	*Apolygus lucorum*	AMQ76482.1	1E-132	100%
OBP31	KT281939	Odorant binding protein 31	*Apolygus lucorum*	AMQ76484.1	1E-104	98%
OBP35	KT281943	Odorant binding protein 35	*Apolygus lucorum*	AMQ76488.1	7E-67	98%
CSP2	KC136233	Chemosensory protein 2	*Apolygus lucorum*	AGD80082.1	4E-85	97%
CSP3	KC136234	Chemosensory protein 3	*Apolygus lucorum*	AGD80083.1	4E-57	100%
CSP4	KC136235	Chemosensory protein 4	*Apolygus lucorum*	AGD80084.1	2E-53	97%
CSP9	MH781728	Chemosensory protein 3	*Apolygus lucorum*	AEP95757.1	3E-70	94%
CSP10	MH781729	Putative chemosensory protein 1	*Lygus hesperus*	APB88037.1	2E-60	95%
CSP12	MH781731	*Putative chemosensory protein 14*	*Lygus hesperus*	APB88063.1	3E-78	91%
CSP16	MN395398	*Putative chemosensory protein 7*	*Lygus lineolaris*	APB88070.1	6E-64	88%
CSP17	MN395399	Putative chemosensory protein 6	*Lygus lineolaris*	APB88069.1	6E-88	82%
OR109	KU958197	Olfactory receptor	*Apolygus lucorum*	AQM56026.1	5E-178	99%
IR21a	MH781745	Ionotropic receptor 21a	*Adelphocoris lineolatus*	APZ81412.1	0	78%
SNMP2a	MH781750	Sensory neuron membrane protein 2a	*Adelphocoris lineolatus*	APZ81422.1	0	81%
Orco	KC881255	Olfactory co-receptor protein	*Apolygus lucorum*	AHC72290.1	–	–

*“–” Orco gene were obtained by gene cloning (Supplementary Figure S5).*

### Expression Profiles of Chemosensory Genes in Legs

FPKM value analysis indicated that the *AlucOBP*9 was the most abundant (FPKM > 8000 in male forelegs, FPKM > 5000 in male middle legs, FPKM > 300 in male hind legs; FPKM > 5000 in female forelegs, FPKM > 3000 in female middle legs, FPKM > 1000 in female hind legs), followed by *AlucOBP*19 (FPKM > 800 in male forelegs, FPKM > 600 in male middle legs, FPKM > 25 in male hind legs; FPKM > 600 in female forelegs, FPKM > 400 in female middle legs, FPKM > 100 in female hind legs), *AlucOBP*3 (FPKM > 800 in male forelegs, FPKM > 600 in male middle legs, FPKM > 30 in male hind legs; FPKM > 500 in female forelegs, FPKM > 300 in female middle legs, FPKM > 90 in female hind legs), and *AlucOBP*26 (FPKM > 300 in male forelegs, FPKM > 300 in male middle legs, FPKM > 100 in male hind legs; FPKM > 200 in female forelegs, FPKM > 200 in female middle legs, FPKM > 200 in female hind legs). In term of *CSP*s, *AlucCSP*9 was the most abundant (FPKM > 2000 in male forelegs, FPKM > 2000 in male middle legs, FPKM > 700 in male hind legs; FPKM > 1900 in female forelegs, FPKM > 1800 in female middle legs, FPKM > 1000 in female hind legs), followed by *AlucCSP*2 (FPKM > 1400 in male forelegs, FPKM > 1600 in male middle legs, FPKM > 600 in male hind legs; FPKM > 1200 in female forelegs, FPKM > 1300 in female middle legs, FPKM > 1000 in female hind legs). FPKM values of both *OR*109 and *SNMP*2a were lower than 10, while FPKM values of *IR*21a were lower than 150 (Supplementary Table S7).

**FIGURE 7:**
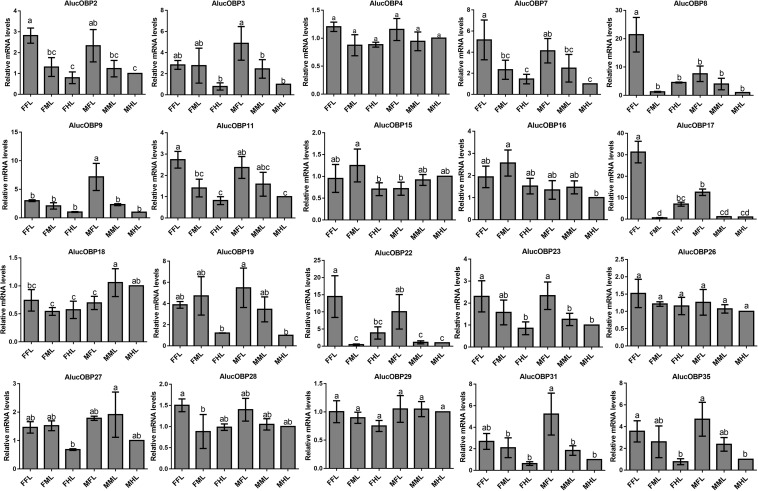
OBP transcript levels in different tissues of *A. lucorum* assessed by qPCR. FFL, female forelegs; FML, female middle legs; FHL, female hind legs; MFL, male forelegs; MML, male middle legs; MHL, male hind legs. The error bars present the standard error, and the different letters indicate significant differences (*P* < 0.05).

**FIGURE 8:**
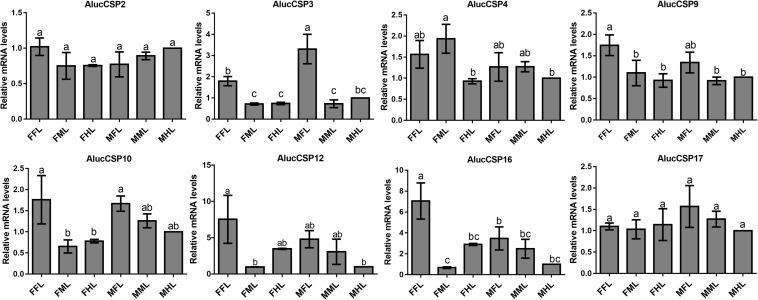
CSP transcript levels in different tissues of *A. lucorum* assessed by qPCR. FFL, female forelegs; FML, female middle legs; FHL, female hind legs; MFL, male forelegs; MML, male middle legs; MHL, male hind legs. The error bars present the standard error, and the different letters indicate significant differences (*P* < 0.05).

**FIGURE 9:**
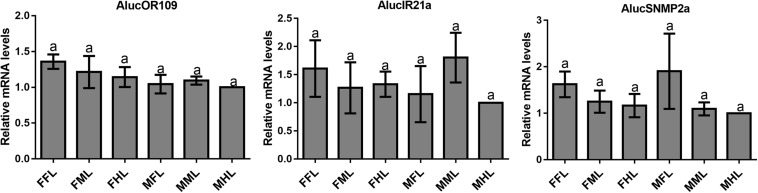
OR, IR and SNMP transcript levels in different tissues of *A. lucorum* assessed by qPCR. FFL, female forelegs; FML, female middle legs; FHL, female hind legs; MFL, male forelegs; MML, male middle legs; MHL, male hind legs. The error bars present the standard error, and the different letters indicate significant differences (*P* < 0.05).

The qPCR results proved that *OBP*9, *OBP*31, and *CSP*3 were highly expressed in male forelegs, while *OBP*2, *OBP*8, *OBP*17, *CSP*9, and *CSP*16 were highly expressed in female forelegs. In details, the expression levels of *Aluc-OBP*2, 8, 11, 17, *CSP*3, 9, 10 and *CSP*16 in female forelegs were significantly higher than that in female middle and hind legs. For males, *Aluc-OBP*9, 17, 31, and *CSP*3 genes showed high expression levels in the forelegs of male, and the gene expression levels were significantly different from those in the middle and hind legs of male. The expression levels of *AlucOBP*8, 17 and *CSP*16 in female forelegs were significantly higher than those in male forelegs, while the expression levels of *AlucOBP*9 and *CSP*3 in male forelegs were significantly higher than those in female forelegs. Moreover, *OBP*18 was highly expressed in male and female hind legs. Additionally, the expression levels of *OBP*4, *OBP*15, *OBP*26, *OBP*27, *OBP*28, *OBP*29, *CSP*2, *CSP*4, *CSP*17, *OR*109, *IR*21a and *SNMP*2a showed no significant difference among forelegs, middle legs and hind legs of both sexes (Figures 7–9).

### Sensilla Types on Legs of *A. lucorum*

**FIGURE 10:**
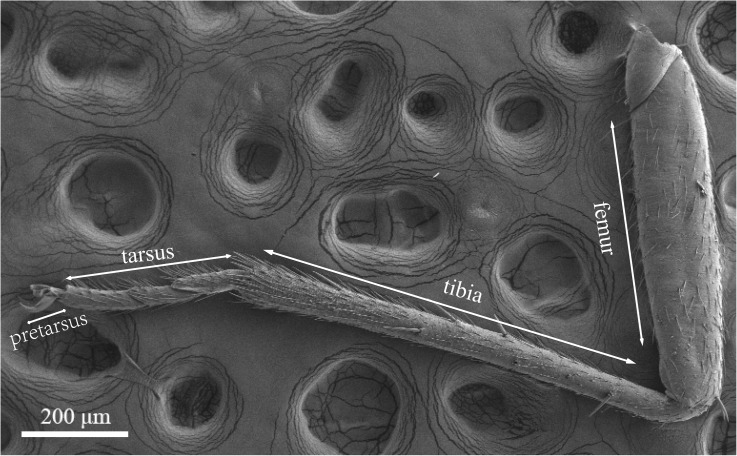
Photograph of foreleg of *A. lucorum* observed by scanning electron microscope.

**FIGURE 11:**
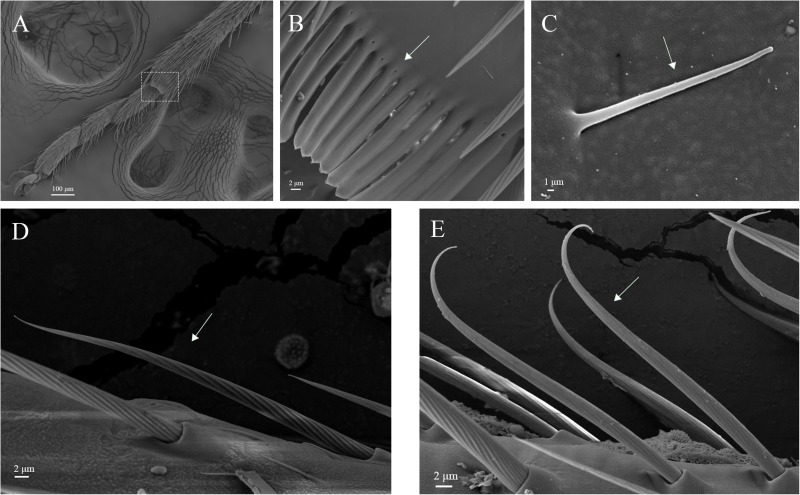
Hair brush, Böhm bristles and sensilla trichodea on legs of *A. lucorum*. **(A)** Brush at the junction of the tibia and tarsus. **(B)** Rows of hair brush with micropores at base. **(C)** Böhm bristles (BB). **(D)** Long straight sensilla trichodea (Str1). **(E)** Long curved sensilla trichodea (Str2).

**FIGURE 12:**
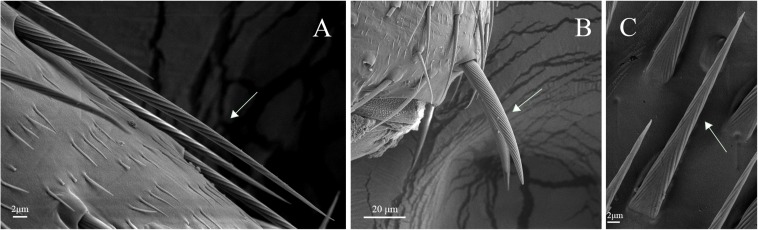
Sensilla chaetica on legs of *A. lucorum*. **(A)** Sensilla chaetica 1 (Sch1). **(B)** Sensilla chaetica 2 (Sch2). **(C)** Sensilla chaetica 3 (Sch3).

**FIGURE 13:**
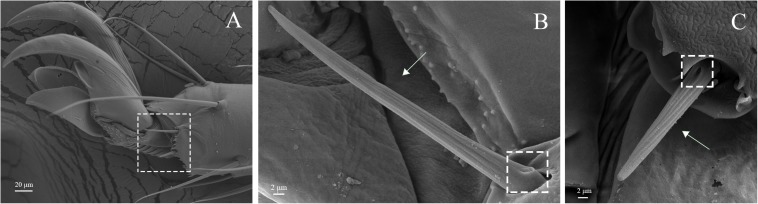
Sensilla basiconca on legs of *A. lucorum*. **(A)** Sensilla basiconca on distal of the tarsus. **(B)** Medium-long sensilla basiconca (Sba1). **(C)** Short sensilla basiconca (Sba2).

Legs of both males and females are consisted of four components: femur, tibia, tarsus and pretarsus (Figure 10). No sexual dimorphism was observed in the legs sensilla types. Scanning electron microscopy results showed that there were four sensilla types on legs of *A. lucorum*: sensilla trichodea (subtypes: long straight sensilla trichodea, Str1; long curved sensilla trichodea, Str2), sensilla chaetica (subtypes: sensilla chaetica 1, Sch1; sensilla chaetica 2, Sch2; and sensilla chaetica 3, Sch3), sensilla basiconca (subtypes: medium-long sensilla basiconca, Sba1; short sensilla basiconca, Sba2), and Böhm bristles (BB) (Figures 11–13). The types of sensilla of forelegs, middle legs and hind legs were the same (Supplementary Figures S2–S4). Additionally, the largest number of sensilla was on hind legs whereas the forelegs had the minimum number of sensilla.

The rows of hair brush with micropores at base were observed in the junction of the tibia and tarsus (Figures 11A,B). The Böhm bristles had smooth surface without pores and base socket, which were distributed in all segments of the legs (Figure 11C).

Sensilla trichodea were divided into two different subtypes and presented in the tarsus of both sexes. The Str1 had a longitudinal grooved surface and softly pointed tip (Figure 11D). The Str2 had a longitudinal grooved surface and gradually curved with blunt tip (Figure 11E). Moreover, two subtypes of sensilla trichodea inserted in the cavities of the legs’ socket (Figures 11D,E). The sensilla chaetica were distributed in the tibia of both sexes. Based on their shapes, sensilla chaetica were further divided into three subtypes: Sch1, Sch2, Sch3 (Figure 12). Each sensillum had a grooved surface and a sharp tip. Sch1 and Sch2 inserted into a socket (Figures 12A,B). However, Sch3 was only distributed in the tibia of hind legs the base of which is directly attached to the cuticle without socket (Figure 12C). The sensilla basiconca were observed in the distal of the tarsus (Figure 13A). Based on their different length, sensilla basiconca were further divided into two subtypes: Sba1 and Sba2 (Figure 13). Each sensillum had a shallow grooved surface and a blunt tip with a pore at base inserting in the cavities of the legs’ socket (Figures 13B,C).

## Discussion

In our previous study, up to 38 OBPs and 109 ORs have been found in the antennae of *A. lucorum* (Yuan et al., 2015; An et al., 2016). Here, we focused on the putative chemosensory genes in legs of both sexes of *A. lucorum* adults. There were 20 OBP genes, eight CSP genes, one OR gene, one IR gene and one SNMP gene identified in legs of *A. lucorum* (Table 3). The total number of chemosensory genes identified in legs of *A. lucorum* was much more than in legs of *A. lineolatus* (Sun et al., 2017). Moreover, the differences in clustering results among six tissue samples indicted that there was a high correlation between female forelegs and female middle legs, male forelegs and male middle legs, female hind legs and male hind legs, respectively, suggesting the similar chemosensory roles of forelegs and middle legs of both sexes, as well as female hind legs and male hind legs (Figure 4).

All the identified AlucOBPs and AlucOR109 in legs are also presented in antennae of *A. lucorum* (Yuan et al., 2015; An et al., 2016), indicating their potential chemosensory function in legs. In *A. lineolatus*, AlinOBP11 was labeled in the tarsal sensilla chaetica and involved in a complicated chemical recognition (Sun et al., 2017). A large number of ORs expressed in insect antennae (Touhara and Vosshall, 2009; Leal, 2013; An et al., 2016). However, we only identified one OR in the legs of *A. lucorum.* It was reported that HoblOR22 specifically expressed in *Holotrichia oblita* legs is a receptor of ligands (Li et al., 2017). We proposed that AlucOR109 in legs may be a receptor involved in olfaction or gustation of *A. lucorum.*

CSPs, another type of odorant carrier proteins, act as chemoreceptors to transport semiochemicals (Wanner et al., 2004). There were five new CSPs identified in legs of *A. lucorum* adults. In a previous study, AlucCSPs showed higher affinities with the secondary metabolites of cotton plants (Hua et al., 2013), suggesting their taste role in insect herbivores. In phylogenetic tree, although CSPs belonging to the same family (CSPs of bug, aphid and plant hopper) cluster together locally, the CSPs from same family segregate into different central clusters. Additionally, some CSPs from mirid bug species showed a very high protein identity and sat at the same branches as orthologous groups (AlucCSP10 and AsutCSP2; AlucCSP13 and LstrCSP1; AluCSP16 and AlinCSP10), which indicated these CSPs may have the same ancestor and differentiate along sex isolation and speciation (Figure 6).

Insect SNMPs play important roles in signal transduction. MmedSNMP2 from *M. mediator* is not only expressed in antennae, but also in head, legs and other tissues, suggesting the multiple roles of MmedSNMP2 (Shan et al., 2019). AlucSNMP2a identified in legs of *A. lucorum* may participate in multiple ecological functions. IR21a and IR25a in *Drosophila* could mediate cool sensing (Ni et al., 2016). Thus, AlucIR21a expressed in legs of bugs may perceive the changes of temperature.

The qPCR results indicated that all the putative chemosensory genes were ubiquitously expressed in forelegs, middle legs and hind legs of adult bugs. Additionally, most genes expressed in forelegs, middle legs and hind legs showed no significant difference such as *Aluc-OBP*4, *OBP*15, *OBP*26, *OBP*27, *OBP*28, *OBP*29, *CSP*2, *CSP*4, *CSP*17, *OR*109, *IR*21a and *SNMP*2a. The *Aluc-OBP*2, 8, 11, 17, *CSP*3, 9, 10 and *CSP*16 showed high expression levels in forelegs of female, and the gene expressions were significantly different from those in middle and hind legs of female (Figures 7, 8), which illustrate that those genes may play more crucial roles in chemosensory behavior in female forelegs than in female middle and female hind legs. On the other hand, *Aluc-OBP*9, 17, 31, and *CSP*3 genes showed high expression levels in the forelegs of male, and the gene expression levels were significantly different from those in the middle and hind legs of male (Figures 7, 8), suggesting that the differentially expressed genes in males may be involved in courtship behavior.

Furthermore, the expression differences of chemosensory genes in forelegs, middle legs and hind legs were compared between males and females. It was found that the expression levels of *Aluc-OBP*8, 17 and *CSP*16 in female forelegs were significantly higher than in male forelegs, indicating their more roles in female forelegs. The expression levels of *Aluc-OBP*9 and *CSP*3 in male forelegs were significantly higher than those in female forelegs, which suggest that these genes may be involved in male courtship in the near distance. The *AlucOBP*18 showed significantly higher expressions in male middle and hind legs than in female middle and hind legs, respectively, which implies AlucOBP18 may be responsible for the senses of touch and taste in male bugs. However, the expression levels of chemosensory genes except *AlucOBP*18 in female and male hind legs had no significant difference, revealing their same roles in both sexes (Figures 7–9). All in all, all the forelegs, middle legs and hind legs of bugs may be involved in close or contact chemical communication. Therefore, chemosensory genes with different expression profiles are associated with different physiological roles in legs of bugs.

Four types of sensilla were observed on the legs of *A. lucorum*, which is consistent with that of *A. lucorum* antennae (Lu et al., 2007). The types of sensilla identified on legs of *A. lucorum* were more than on legs of *A. lineolatus*. Moreover, the types of sensilla on legs of *A. lucorum* showed no sexual dimorphism. The similar results also appeared in *A. lineolatus* and *Adelphocoris suturalis* (Sun et al., 2017; Lu et al., 2009). In *L. lineolaris* and *A. lineolatus*, sensilla trichodea were involved in perception of olfactory stimuli (Chinta et al., 1997; Sun et al., 2013a). In lepidopteran species, sensilla trichodea are reported to respond to female-produced pheromones (Leal, 2005). Additionally, sensilla trichodea in lepidopteran females detect their own sex pheromone and lead to aggregating to increase chance of mating, moving away from calling females, or stimulating oviposition (Birch, 1977; Saad and Scott, 1981). We proposed that sensilla trichodea on legs of *A. lucorum* may play a vital role in olfactory behavior. Böhm bristles were distributed in all segments of legs of *A. lucorum* (Figure 11C). However, in antennae of *Adelphocoris fasciaticollis*, Böhm bristles are found at the scape and pedicel segments, especially abundant in the joints of the antennal segments (Sun et al., 2013b). Three types of sensilla chaetica were observed on legs of *A. lucorum* (Figure 12). In *A. lineolatus*, AlinOBP11 located in sensilla chaetica show highly binding abilities to the bitter substances catechin and quercetin (Sun et al., 2017). Thus, sensilla chaetica may be involved in the perception of bitter substances. Additionally, sensilla basiconca enriched in micropores contain abundant nerve cells, suggesting their potential chemoreception roles (Ochieng et al., 2000; Bleeker et al., 2004). In *A. lineolatus*, AlinOBP1 located in sensilla basiconca could bind the volatile compounds (Gu et al., 2011). Moreover, AfasOBP11 was located in the two types of sensilla basiconca in *A. fasciaticollis* (Li et al., 2019). In addition, AlinOBP13 expressed specifically in basiconic sensilla had strong binding affinity to terpenoids (Sun et al., 2014). In this work, sensilla basiconca with pores were found on legs of *A. lucorum* (Figure 13) and may be involved in olfactory perception. To sum up, we proposed that sensilla on legs of *A. lucorum* could play dual roles in gustation or olfaction.

## Data Availability Statement

The datasets generated for this study can be found in the PRJNA590311.

## Author Contributions

YJZ, ZL, and SL conceived and designed the experimental plan. ZL, YYZ, and XA performed the experiments. QW and AK analyzed the data. ZL and SG drafted the manuscript. SL and YJZ refined and approved the final manuscript.

## Conflict of Interest

The authors declare that the research was conducted in the absence of any commercial or financial relationships that could be construed as a potential conflict of interest.

## Footnotes

1 https://github.com/trinityrnaseq/trinityrnaseq/wiki

2 ftp://ftp.ncbi.nlm.nih.gov/genomes/Gossypium_hirsutum
