# Supplementary material for: Identification of Leg Chemosensory Genes and Sensilla in the Apolygus lucorum
Source: Front Physiol. 2020 Apr 15;11:276. doi: 10.3389/fphys.2020.00276 (PMC7174674; doi:10.3389/fphys.2020.00276)
Supplement: Supplementary file 1 [file Table_1.DOCX]

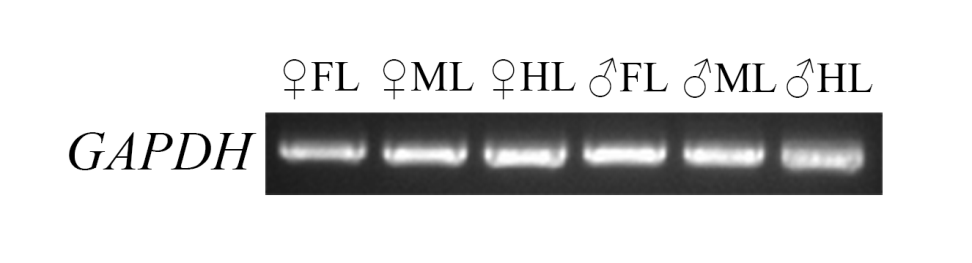


**FIGURE S1** The stable expression of *GAPDH* in different tissues of *A. lucorum* assessed by RT-PCR. ♀, Female; ♂, Male; FL: Forelegs; ML: Middle legs; HL: Hind legs.


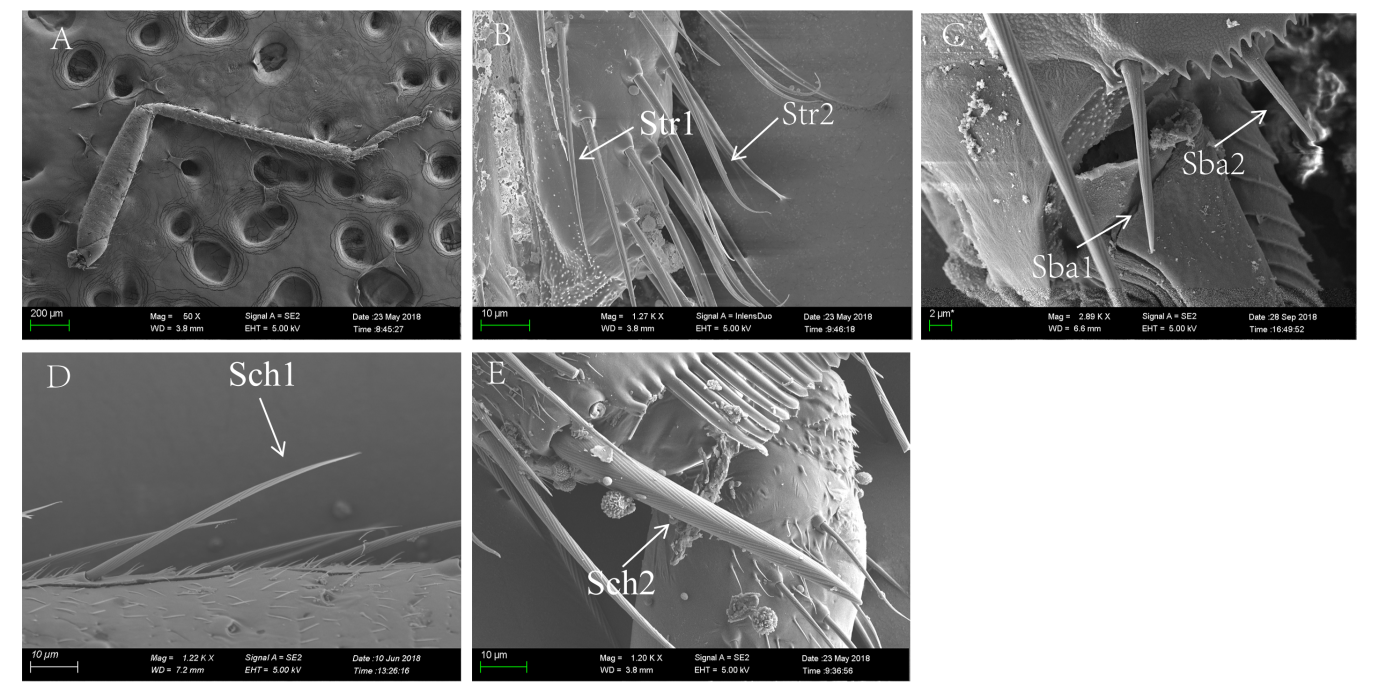


**FIGURE S2 Sensilla on forelegs of *A. lucorum.***

**A:** Foreleg of *A. lucorum*. B: Long straight sensilla trichodea (Str1); long curved sensilla trichodea (Str2). C: Medium-long sensilla basiconca (Sba1); short sensilla basiconca (Sba2). D: Sensilla chaetica 1: Sch1. E: Sensilla chaetica 2: Sch2.


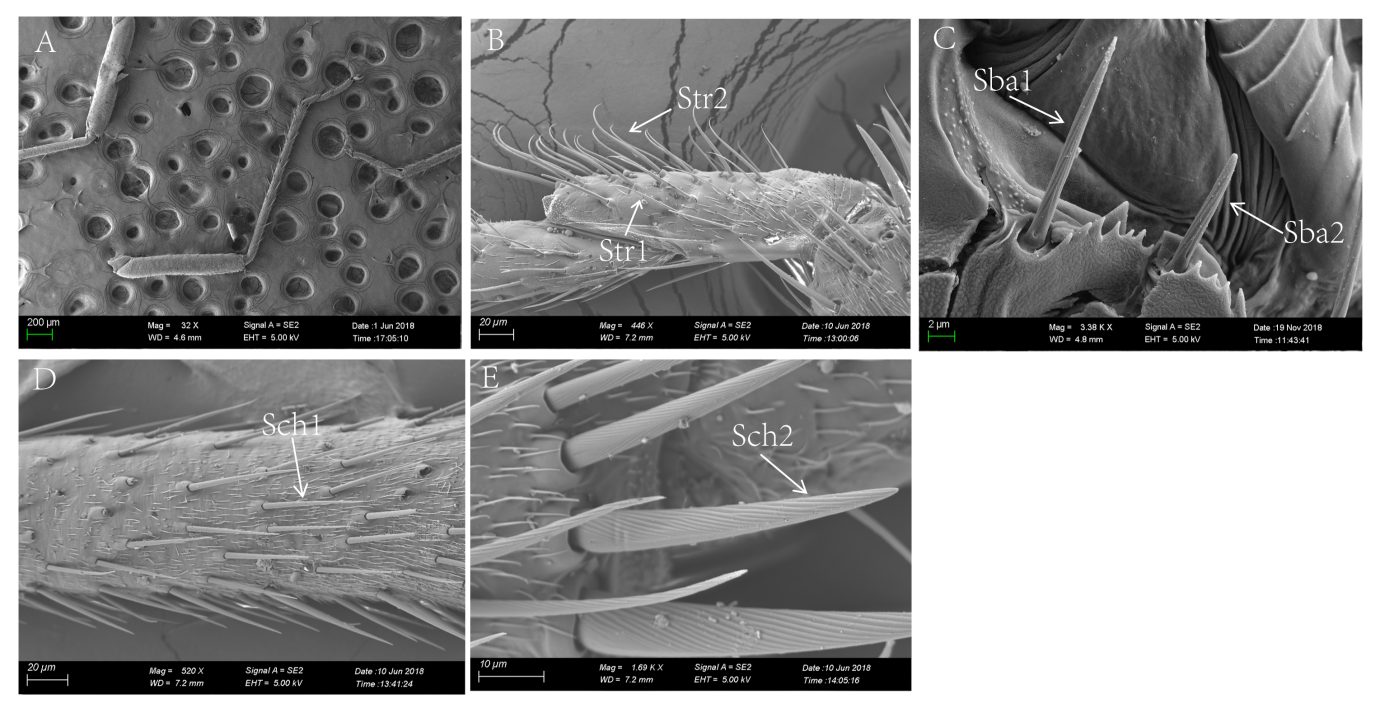


**FIGURE S3 Sensilla on middle legs of *A. lucorum.***

**A:** Middle leg of *A. lucorum*. B: Long straight sensilla trichodea (Str1); long curved sensilla trichodea (Str2). C: Medium-long sensilla basiconca (Sba1); short sensilla basiconca (Sba2). D: Sensilla chaetica 1: Sch1. E: Sensilla chaetica 2: Sch2.


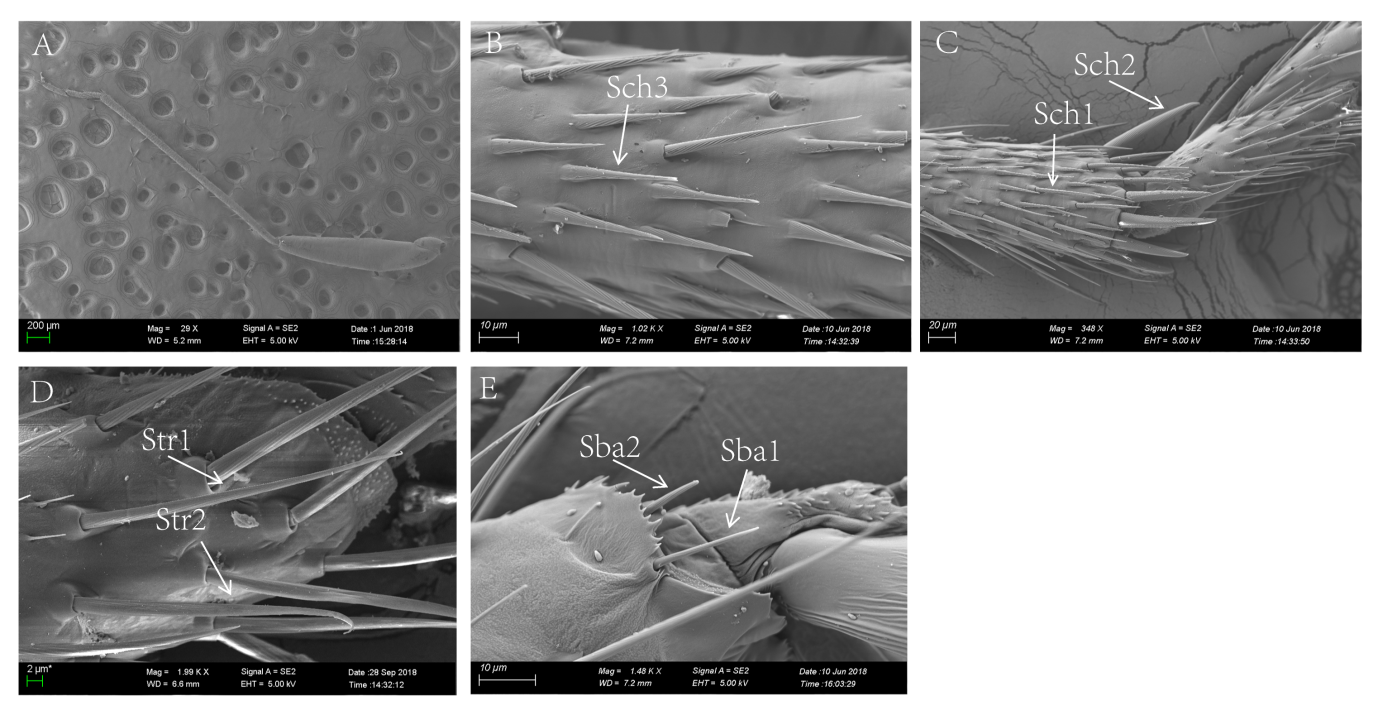
 **FIGURE S4 Sensilla on hind legs of *A. lucorum.***

A: Hind leg of *A. lucorum.* B: Sensilla chaetica 3: Sch3. C: Sensilla chaetica 1: Sch1; Sensilla chaetica 2: Sch2. D: Long straight sensilla trichodea (Str1); long curved sensilla trichodea (Str2). E: Medium-long sensilla basiconca (Sba1); short sensilla basiconca (Sba2).


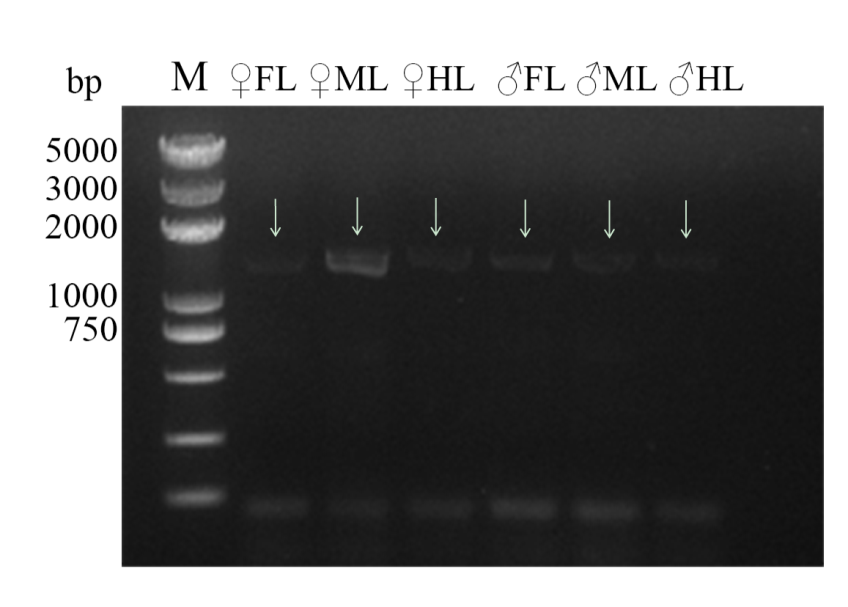


**FIGURE S5** The *Orco* in different tissues of *A. lucorum* assessed by PCR. ♀, Female; ♂, Male; FL: Forelegs; ML: Middle legs; HL: Hind legs. The full length of *Orco* is about 1416 bp.

**Table S1** Primers used in gene cloning and sequencing.

| **Genes** | **Forward (5'-3')** | **Reverse (5'-3')** |
| --- | --- | --- |
| AlucOBP2 | AGCACGGGAAAAATGAATCTATG | ACAGGAAGAGCTTCTCAAACGC |
| AlucOBP3 | TGGATTCATCAATGTTCTCCTCTG | CGGGTGAAACTTGAATCTAATGTT |
| AlucOBP4 | ATGGAGGTTGCAGCTTGCC | CTAGGGAGACGTCCCGCAA |
| AlucOBP6 | GAAGGACTTCAGACGGCCATA | TCATTTTCAGTTCTTTTTGATTCCT |
| AlucOBP7 | ATCTACAAGCGACATCATGAACC | CGGTGTGTTTTCAGAAATCTGG |
| AlucOBP8 | AATACTTGGTGTCACCAGGGGA | TCTGACTTAGTTTTTCATGGAACAAT |
| AlucOBP9 | ATGAAGTCTTTCGTAGGTTTGATCT | CCGTTCTGGTTCAGCGTCTT |
| AlucOBP11 | CTCGACATGGGATCTCAGTATGA | GTTCATGATGTGTTCTCGAATATTG |
| AlucOBP15 | CTCACTACTTCGACGATCATGATG | AAGTCACTCCTTACTCCTTTAAAGG |
| AlucOBP16 | ACCTCTTCGTCTCATCATGAAGAG | ACCCTCATGTTGAGCCTGGT |
| AlucOBP17 | CTCTCTACCCAGCCGAAATGAG | TTATGTAATTAGATCACCATGAAGTTCA |
| AlucOBP18 | CCGTCTCCCTCAACCAATCAT | AGATCGGGGTTTTACGCAGAT |
| AlucOBP19 | AAAGTGAACGTCATGAACTCTCGT | ATCAGGAGGCGTTAGCAAAGTC |
| AlucOBP22 | ATACCAACGAAGTCACACTATGTCTCT | GGTGAAGTGTGTCTCAGTTAAGACC |
| AlucOBP23 | GTGATCGTTCAAACATGTACGTCTT | GGTTCATCGGTGGGGGAAT |
| AlucOBP26 | ACAGCAACAAACATGAATCCCAC | CAGTATTTTTTCTATATGGTGAAGAATTC |
| AlucOBP27 | GGCAGGATGGCCCGCA | GGAAAGTATTATTGAGAAATGCCTG |
| AlucOBP28 | AACGACATCTTGAAATCATGATCAT | TTTGCAGACATTATTTCCTCTGAAT |
| AlucOBP29 | TCACACCGAATCATGAATCGT | GTTGTTGATTGGTAACTGATCACTGA |
| AlucOBP31 | CTCCCACAACATGTTCACCTCT | TGAAGTGAAGTTCGAATCAATGTTT |
| AlucOBP35 | CATGATCAACGTCGTTTTCGTT | AGAAAACATTAACCCCGCATT |
| AlucCSP2 | ATGGCCAGCAAATTGTCGGTAG | GTACTCGATGGGTTTCCCCTCTTCG |
| AlucCSP3 | ATGAAGGTTGCCGTGTTGGTTTTAC | AGCGCTGATGGCTTTGGCTTCTTTC |
| AlucCSP4 | ATGAAATTCGTAGCGGCCCTCTTCG | AACGTTGATTCCGAGTTTTTTGGC |
| AlucCSP9 | TGAACTTGGGATTCCTTTT | ATTCGGTGGTTTTTCTTGA |
| AlucCSP10 | CTTATCAACAATTCGATCCC | TCTATAGCAGTTACTTTCCCTC |
| AlucCSP12 | ATGGTGTGGTTACTTCTGTG | AGACTCGATGAATTCCTTAAC |
| AlucCSP16 | ATGCGGATAATCTACGCCTTCCTG | AGACTCGATGAATTCCTTAAC |
| AlucCSP17 | ATGTTGATGAGAATCGTGATTAC | AATAACTGGAGTAGTTAAATTATGTCTAT |
| AlucOR109 | TGATGCTTCGTTTACTTCGC | CACCAGTTTAATCGTTCGG |
| AlucIR21a | CTCGTAGACTTCTTGAACACA | GATTATGAAATGCTAGGAGAGT |
| AlucSNMP2a | AACACCGCAACACTCTCAAG | GCAACCAATCAAAGAATCACCT |
|  |  |  |

**Table S2** CSP sequences used in the phylogenetic tree construction.

>AlucCSP1

MLKVLVLLAAVVCCVSAAATYTSKYDNIDLDEILSNTRLYKKYFDCLANKGKCTPDGKELKESLPDALKTNCAKCTKKQQEGTDKVLRHVLKNKPNDYKVLESIYDPTGIYRKKYEIEAEKRGIKLPGSH

>AlucCSP2

MVGKLSVVLLIGAVGMVLAADKYTDKYDNIDVDEILGNQRLYQKYFDCIQGKGKCTPDGAELKKNIPEALQTDCAKCSEKQKAGVEKVLRHLINEKPEDYKVLEEQFDPEGVYRKKYEHLKKKVEEGKPVEY

>AlucCSP3

MLKVLVLLAAVVCCVSAAATYTTKYDNIDLDEILSNQRLYKKYYDCLANKGKCTPDGKELKEALPDALKTNCSKCSKKQQEGTDKVLRYVLKNKPNDYKVLENIYDPSGNYRKRYEDEASKRGIKLPGSH

>AlucCSP4

MVSKLSIVLLIGALADVWASELYTDKYDNIDVDEILGNQRLYQKYFDCIQGKGKCTPDGAELKKNIPEALQTDCAKCSEKQKAGVEKVLRHLINEKPEDYKVLEEQFDPEGVYRKKYEHLKKKVEEGKPIEY

>AlucCSP5

MVGKLSVVLLIGAVGMVLAAELYTDKYDNIDVDEILGNQRLYQKYFDCIQGKGKCTPDGAELKKNIPEALQTDCAKCSEKQKAGVEKVLRHLINEKPEDYKVLEEQFDPEGVYRKKYEHLKKKVEEGKPV

>AlucCSP6

MVSKLSIVLLIGALADVWASELYTDKYDNIDVDEILGNQRLYQKYFDCIQGKGKCTPDGAELKKNIPEALQTDCAKCSEKQKAGVEKVLRHLINEKPEDYKVLEEQFDPEGVYRKKYEHLKKKVEEGKPIEY*

>AlucCSP7

MVSKLSIVLLLGALADVWAAELYTDKYDNIDIDEILNNDRMYKNYFNCVMGNGKCTPDGLELKAKIPEALQTECAKCSDKQKKGAEKVLRFIINQKKDDYKLLEEKFDPEGVYRKKYEAQKKLAEEGKPIEY

>AlucCSP8

MLKVLVLLANAASTYTTKYDNIDLDEILSNQRLYKKYYDCLANKGKCTPDGKELKEALPDALKTNCSKCSKKQQEGTDKVLRYVLKNKPNDYKVLENIYDPSGNYRKRYEDEASKRGIKLPGSH

>AlucCSP9

MLKIFVLLAAAVCLANAASTYTTKYDNIDLDEILSNQRLYKKYYDCLANKGKCTPDGKELKEALPDALKTNCSKCSKKQQEGTDKVLRYVLKNKPNDYKVLENIYDPSGNYRKRYEDEASKRGIKLPGGH

>AlucCSP10

MNTSALLKVGILLGCIAACLAAETRSSVSDEALEAALKDKRYLTRQLKCALGEGACDPVGRRLKTYAPLVLRGACPKCTPSEVRQIQQVLSHIQRHYPKEWAKILKQYAGQ

>AlucCSP11

MKIFLFSLLLVSVATVVLCAEVYPDKYDSIDLDEILSNQRLYQKYFDCVMGKGKCTPDGTELKDKIPEALQNECAKCSEKQKKGAEKVLRFLITEKKDDFKLLEEKFDPEGKYRKKFGELKKKIEEGKPVTV

>AlucCSP12

MVWLLLCFLLFAAGRAEDMTQEDMEFYTRVFEEVDPDFILDNERILTSYLKCFYSEIECNAHAEVVKKSIPNVLATVCGRCSDKQKGIFKYSLNKFIPAHPKDWERILSIYDPTGEAWPKVKEFIES

>AlucCSP13

MKILLVLGALVAAVASQGGLPFLTPSEVNRLISDSNYVKNQINCILGKAKCDSLGNQLKLAIPEVLGRNCKNCDAQQAANARKVTDFMRNNFPAEFGQILKRYRIRQSHSYGR

>AlucCSP14

MRRGEGTMIRLTVVLLTIASVARTGVCTRLPYSTHYDYIDVDQVLNNSRLYTKYFECLMGQGKCTPEAKELRDKLPEALQTNCGICSERQAKESHKVIRFLINQRPEDFKMLEAKYDPSGLYMKRYQEEMKLNGAFS

>AlucCSP15

MLIFVVFGVSSLFVGLEGAPLQYSDTRYDDVELTSILNNDELYIKLFQCLIGRGKCTPDWEILKDAVPGALLDNCEKCTAKEKFGTKTLLSHLVHEKPSDMRILEGEFDPDGSYRKELEKEEKETNDINRKRSALKEEVEFLNKVKRLIK

>AlucCSP16

MRIIYAFLVVLACGLASSEMTEEEFYEKVFEEVDPDFILDNERILTTYLKCFYNEIECNVHAEVVKKSIPNVLATVCGRCSDKQKGIFKYSLNKFIPAHPKDWERILSIYDPTGEAWPKVKEFIES

>AlucCSP17

MLMRIVITIAAVLTVSLALVTREMREREFFRQLEGINVDSILINRRLIDKYIKCLLKTGKCDPTMKDLRIALPLILEHLCETRCSERERQNLRKLFLYIRTNRAQEWERLAKLYDPKGAYKANVDAFIENRPRPTMITSSAIRETVAPTIFTTTTTTTTTTTRAPTAPTPQRQSIRATRNALYIAMRRFAMYATTPRKRNRHNLTTPVI

>AlinCSP1

MLKVLVLLAAVVCCVSAAATYTSKYDNIDLDEILSNTRLYKKYFDCLANKGKCTPDGKELKESLPDALKTNCAKCTKKQQEGTDKVLRHVLKNKPNDYKVLESIYDPTGIYRKKYEAEAEKRGIKLPGSH

>AlinCSP2

MKVAVLVLLCVGAALSAEVYTSKYDNIDVDKILSNDRILTQYIKCLMEEGNCTNEGKELKKTLPDALASGCTKCSEKQKAQTEKVLRHLSKNRPRDWARLKTKYDPKGEYSKKYEKEAKALTS

>AlinCSP3

MISKLSMVLLIGALADVWAAELYTDKYDNIDIDEILNNDRMYKNYFHCVMGNGKCTPDGLELKAKIPEALQTECAKCTDKQKKEVEKVLRFIINQKKDDYKLLEEKFDPEGVYRKKYEAQRKLVEEGKPVEY

>AlinCSP4

MRIILSAFLVAMACSLATCEMTEEEFYTKVFEEVDPDFILDNERILTSYLKCFYNEIECNAHAEVVKKSIPDVLATVCGRCSDKQKSIFKYSLNKFIPAHPKDWEKILSIYDPSGEAWPKVKAFIES

>AlinCSP5

MGHLTIVLLAAAFEVLTGGRAYTTHYDYIDVDQVLNNTRLYTKYVECLLGQGKCTPEARELRDKLPEALQTNCAKCSERQASESHRVIRFLIQNRPEDFKLLEAKYDPSGLYFKRFEEETKRNVSLS

>AlinCSP6

MFYKLSVVVLMGILAGVWAADKYTDKYDNIDIDEILTNERLYKKYFDCIQGTGKCTPDGIELKEKIPEALKTECAKCNEKQKAGVEKVMRYLITKKPEDFKILEDKFDPEGVYRKKYEAQRKLVEEGKPVEY*

>AlinCSP7

MNYKLSVILLIGVLASVWAASTYTDKYDNIDLDEILTNERLYKKYFDCIQGKGKCTPDGTELKEAIPDALKTECAKCNAKQKAGVEKVLRHLLTKKAEDYKILEDKFDPEGVYRKKYEAQKKLADEGKPIVL

>AlinCSP8

MDYKLSVMLLMGVLACAWAADKYTDKYDNIDIDEILNNERLYKKYFDCILGNGKCTPDGTELKETIPDALKTACAKCNDKQKAGVEKVLRHLLTKKAEDYKILEAKFDPEGVYRKKYEAQKKLAEEGKPIAL

>AlinCSP9

LAVVTREMREREFFRQLEVINVDSILINQRLIDKYIKCLLKTGKCDPIMKDLRIALPLILGHLCEARCSEK*

>AlinCSP10

MRSNFINESIPDVLATVCGRCSDKQKSIFKYSLNKFIPAHPKDWEKILSIYDPSGEAWPKVKAFIES*

>AlinCSP11

MKVFFSGLLLVCMASVSLCADEYTDKYDSVDLDEILNNQRLYQKYIDCVMGKGKCTPDGALLKEKIPEALQNECAKCSAKQKKGAEKVLRFLINEKADDYKALEEKYDPEGTFRSKYEEQKKNLKEGKPLSV

>AlinCSP12

MMIIIVFGISALLVVVEGAPLQYSDTRYDDVELTTILSNDELYIKLFQCLIGRGKCTPDWEILKDALPGALLDNCSECSNKQKFGTKTLLAHLVHERPSDMRLLEGEFDPDGSYRKELEKEEKESNDINRKRSANLEEVEILDKIKRIIK

>AlinCSP13

MKFVAALLVASVAVLAVEAANQYTTKYDNIDLDDILKNQRLYKKYFECLTGNGKCTPDGKELKEHLPDALKTGCSKCSEKQRAGSEKVIKHLLKNKPQDYAVLEKIYDPSGIYKKKYEAEAKKLGINV

>AlinCSP14

MNSAIVLCVVALAGMVLARPDDTYTTKYDNVDLDEILGNDRLLVPYIKCTLDEGKCAPDAKELKEHIREALENGCAKCTDKQKEGTRRVIAHLIKHKNADWQKLKAKYDPEGKYTHKYEKELEEVQH

>AlinCSP15

MKLIVAVALLCVVAESWAASTYTDKWDNINVDEILESQRLLKAYVDCLLDRGRCTPDGKALKETLPDALENECSKCTDKQKSGSDKVIRHLVNKRPEMWKELSAKYDPNNIYQDRYKDKIEAVKGQ

>AlinCSP16

MLPFYVFSLCAVFVACQETYTSKYDNVNVEDALKNDRLYKAYFNCLADRGPCTREGNMLKEALPDGLRNNCSLCTDPQRRGTHQVIRFLFKYRPEDMKLLEEIYDPEGIYKTKYAEERKKLME

>AlinCSP17

MDYKFLVVMQMGVISSVCAAGPYTDKYDNVNLDEVLNNERLYRNYFNCLQGKGKCTLDGAILKEIIPSALKTDCALCSVRQKKGAEKVLIFLITKKPDDFKILEDKF

>AlinCSP18

MIWILLVAVSMTTSLAEEESIDYYRVFEEIDPDLILDNERILQTYLKCFYGEGPCNTHAQLAKESIPDVLA

>AlinCSP19

MVYKSSVVFLLMGTVAYVWGEKYTEQYDDINLDNILTNERLYRIYFKCILSKGKCTPEGEVLKKAIPDALK

>AlinCSP21

KELKEHIKEALENECGKCTEAQKKGTRRVIGHLINHEADFWNELTAKYDPERKYTTKYEKELKEVKA*

>AgosCSP1

MNILTIFCYVTVMCDTQVKPAVSAQRLQSVNQNVTPTNDGRKTIRETSSYPTRYDYIDIEAVMNNERIIKILFNCVMSRGPCTREGLELKRIVPDAIQTECAKCNERQRKQAGKVLAHLLQYKPEYWKMLVQKFDPNNVYLRKYMADNDDDEKLSLQKLSNDTTKKKRNI

>AgosCSP2

MAHLNLFVVLIASLIYFTSAAEEKYTTKFDNFDVDKVLNNNRILTSYIKCLLDEGNCTNEGRELKRVLPDALKTDCSKCTDVQKDRSEKVIKFLIKNRSTDFDRLTAKYDPTGEYKKNLEKFEKERASAKPLKA

>AgosCSP3

MDSRIAVVCVVLAAFAVDQTVGAPQKDAVAASGPAYTTKYDHIDVDQVLASKRLVNSYVQCLLDKKPCTPEGAELRKILPDALKTQCAKCNATQKNAALKVVDRLQKDYDAEWKQLLDKWDPKREHFQKFQQFLAEEKKKGFTKF

>AgosCSP4

MHCKVLIALCCVAVYAVQASPAGTATAAAVSADDEIKDFPAYMKRFDKLNVEQVLNNDRVLASHLKCFLNEGPCVQQSRDLKRVIPVIANNGCNGCTERQMTTIKKSLNFLRTKKPTEWARLVKIYDPSGTKLNKFLDA

>AgosCSP5

MIKLILAIAFCVSITMTVVQTAPAKYTTKYDNVNIDEILNNDRLVASYFKCLMETGKCTPEGEEIKRWLP

EAIENKCEDCSEKQKLGSEKIIKFLFEKKNDMWKQLEAKYDPQGTYRQRYAEEAKKLNINV

>AgosCSP6

MSRSSSSVTMKVFVIAICVCAALARPEDVKVENKPAVIKSETLAAPLPTNIVKRATDTIQLDSSLPNVSE

DVLDKALSDRRFVQRQLKCATGEGPCDPIGRKIKAHAPLVLRGMCVKCSQSEIKQIQRVMSHIQKNYPKEYTKMLKQYQSGF

>AgosCSP8

MNNIIMNNSRGRYGIFSLLAVTIAAIMLVHQPATVRCADGGIITPQQQQQQTMMFTAPTGYYVSTYDHID

VGRLLRNNKVVSGYVKCFVNEGPCTPDGKLVKAYLLPEIIRTVCGKCTPRQKDMARMVLKHIYTYRQADFEKIMQIYDTDGKRNEILAFMNH

>AgosCSP9

MSAFCLNSFILMTMITVIVTHATFTRSTKFDDRTGIDIHLVKRDTDDVNDDENSVESDEGFFYRFTHFFQ

DSSDKEDDDDDEKKPDFITTFDIFKLLDEEYAMQQFYCVINEDPCDEVGMRLKATIPEEINRNCERCTST

ERNNIRRILNYVKKHYPQFWKRVEPIYKKKI

>AgosCSP10

MINTRPRKLVRCIRGVSISVAKGDDAVNAENKDDDSHLVNREEIQRYMSMMEKINIDQMLNNTRLMSNNVKCFLNEGPCTAHLREMKKMVPMLVKDSCSSCTKEQKIMMKKAMDAVKARRPNDYEKLSKFFDPEGKYEKKFLENLNESK

>AsutCSP1

MLPFYVFSLCAVFVACQETYTSKYDNVNVEDALKNDRLYKAYFNCLADRGPCTREGNMLKEALPDGLRNNCSLCTDPQRRGTHQVIRFLFKYRPEDMKLLEEIYDPEGIYKTKYAEERKKLME

>AsutCSP2

MGHFPPVFSLSPVLLVASLHTMNTSTLLKIAFLLGCVAACLAAETRSSVSDEALEAALKDKRYLTRQLKCALGEGACDPVGRRLKTYAPLVLRGACPKCTPSEVRQIQQVLSHIQRHYPKEWAKILKQYAGQ

>AsutCSP3

MKFVAALLVASVAVLAVEAANQYTTKYDNIDLDDILKNQRLYKKYFECLTGKGKCTPDGKELKEHLPDALKTGCSKCSEKQRAGSEKVIKHLLKNKPQDYAVLEKIYDPSGIYKKKYEAEAKKLGINV

>AsutCSP4

MRIILSAFLVAMACSLATCEMTEEEFYTKVFEEVDPDFILDNERILTSYLKCFYSEIECNAHAEVVKKSIPDVLATVCGRCSDKQKSIFKYSLNKFIPAHPKDWEKILSIYDPSGEAWPKVKAFIES

>AsutCSP5

MDYKFFVVMQIGVISSVCAAGTYTDKYDNVNLDEVLNNERLYRNYFNCLQGKGKCTLDGAILKEVIPSALKTDCALCSVRQKKGAEKVLIFLITKKPDDFKILEDKFDPEGVYRKKYEAQRKLVEEGKPIH

>AsutCSP6

MVCKLFAVVLMGILAGVWAADKYTDKYDNIDIDEILTNERLYKKYFDCIQGIGKCTPDGIELKEKIPEALKTECAKCNEKQKAGVEKVMRYLITKKPEDFKILEDKFDPEGVYRKKYEAQRKLVEEGKPVEY

>AsutCSP7

MVSKLSMVLLIGALADVWASELYTDKYDSIDIDEILNNDRMYKNYFNCVMGNGKCTPDGTELKAKIPEALQTECAKCSDKQKKGVEKVLRFLIKEKKDDYKLLEEKFDPEGVYRKKYEAQKKLVEEGKPIEY

>AsutCSP8

MDYKLSVMLVMGVLACAWAADMYTDQYDNIDIEEILTNERLYKKYFDCIIGNGKCTPDGTELKETIPDALKTACAKCNDKQKAGVEKVLRHLLTKKAEDYKILEAKFDPEGVYRKKYEAQKKLAEEGKPIVL

>LstrCSP12

MFKNLLVVCLLVAAVSAKPKPAEKKNTTKYDNIDLDEILNNQRLFDNYYKCLLGAKCTPDGQELKEALPDALATACSKCTEKQRVGTEKVIRHLIEKKPTEYAELEKKYDPQGTYKRKYQAEAIKRGIKV

>LstrCSP11

MKFLYFTVFGCALVMFTSAIPEIIFTSAMPQKTYSTMYDHINVNNILKNDRLFNRYFTCLTKRGGCTPEG

KLLAAAILDALETSCANCSNEQRKLARQVIQYL

>LstrCSP10

MYDHINVYNILKNERLFNRYFTCLTKKEGCTPEGKLLAAAILDALETSCANCSNEQRKLAEQVIQYLYFNKRDKFDELAMIYDLEGVFQEYHIAEYLVSGSWMPDFRKLPPV

>LstrCSP9

MSEVLVMILIFMLLAGREQRLQQQQQQQQQPQPQQQNVDNIEMSIYDKMFENMDVNSLLKNHRLVDSYLK

CFLNEGSCTHIGHEVKMMIPEVIRSKCATCGENQMRALKAGLRLFIALRPDDWKRFLDVYDPDRTEWPHIKAFMEYDD

>LstrCSP8

MKSPCLLSVSCCILVLVASSASAAPKEKDPERKALYRLEYIDIEKVLDNNRMLTNFIRCFLRKGPCSPEA

RDFRKLLPKLAKTMCSDCSPRQRFIIKKVFKHLMEERPKEWELLMDRFDPQRKYAERLDTFMVDMTTRATPTTTTTTIPTTTTPMSSTTQRIIEILRTSTEMSNESRP

>LstrCSP7

MIQRTQGFNSIVVLLLIKLTVLSMVLASTHAPAPTPTPKVETKATEAAKSSSKDEIPDQTFDRYINNERY

MLQQYECLMGNKPCDHVGRKLKAAVPLVVRGLGCPKCSPREEEQMKRIVSHVQRSYPDKWQKLIRKYGQ

>LstrCSP6

MQASSLAMLLIAVWVLSPRRPLSGGFAGVHAQQSKNTRYTTRFDSIDVEVILKNERIFKRYMDCLLDKGRCTPEARELKRLLPEALKTECLKCSEVQRRQGAKVMAFIIKNKRPYWDLLLAKYDPQGVFRAKYKYNDQNIEAVLKQLEREQQGLYGTYSNPTNTTTVNSASSRK

>LstrCSP5

MLKFKLTLLVMASAFFSVDGGKLYKDRYTTKFDKIDLDEALNNQRLFESYLKCLMGDKCSPDGYELREALPDALATACAKCSEAQKAGTEKVIRFLIEKRPKEYALLEKKYDPEGVYRDKYKPIAEEKGIKI

>LstrCSP4

MLWAAKFIVFPLIFCVLQVWSAPADEKYSDIDFESILANRRVLSSYVKCLTDKGPCTPQGKELKKIVPEV

IQTSCTKCSPQQKKVVRNVITTMQSKYKDQWDLVVNKYDPKKQRAGELKAFLAGTD

>LstrCSP3

MKLALFCCLLGLVIAVSAEKYTTKYDHINVEEILNNERLFNSYYKCLMGGKCTPDGLELRTHLPDALRTNCSKCSEKQKEFSDKVIRYLIDNKPEEFAALTKKYDPEGIYKTTFGPQFKKDNTTTNQ

>LstrCSP2

MRCLLLVAVICAAFIAAAQADEANKYTSKYDNIDIDKILKNDRVLSQYIKCLMGEGSCTQEGRELKRLLPDAIQSNCSKCSEKQRQASVKVMRHLRQSRVRDWNRLLDKYDPQGDKRKNLKLD

>LstrCSP1

MASVSSATLTAAALLALLALQLTAAQNFNEADIARMLNDSGLVQRQISCILGEAACDNIGNMLKLAIPEVLKRNCRSCNAQQASNARRLISFVQANYPAQWQRIQSRYVG

>SfurCSP9

MRCLLLVAVVFAAFIAAARADEANKYTSKYDNIDIDKILKNDRVLSQYIKCLMGEGSCTQEGRELKRLLPDAIQSNCSKCSEKQRQASVKVMRHLRQSKERDWNRLLDKYDPQGDKRKNLKLD

>SfurCSP8

MLKFTLTLLVLAVVSVNCGKLYKDRYTTKFDKIDLDEALNNQRLFESYLKCLMGDKCSPDGYELREALPDALATACAKCSDAQKAGTEKVIRFLIEKRPKEYALLEKEYDPEGIYRDKYKPIAQEKGIKI

>SfurCSP7

MRASKASSLVAVLLIAVWGFTGVQAQQKSKDTRYTTRFDSIDVEVILKNERIFKRYMDCLLDKGRCTPEARELKRLLPEALKTECLKCSEVQRRQGAKVMGFIIKNKRPYWDLLLAKYDPQGIFRAKYNYNENNIEGVLKQLEREQQGLYGTYSNTTNTTNTVNSTSTRK

>SfurCSP6

MKLALFCCLLGLVAAVSAQSEKSEKPEKYTTKYDYINVDEILSNDRLFNSYYKCLMGGKCTPGGPELRTHLPDALQTNCSKCSEKQKEFSDKVIKHLMDNKPEEFSALVKKYDPEGIYKDAFKPKHNQ

>SfurCSP5

MSEILVTSLIFMLLAASELGLGQQQQTQKPQQQNVDNIEMSIYDKMFENMDVNSLLKNHRLVDSYLKCFLNEGSCTHIGHEVKMMIPEVIRSRCATCGENQMRALKAGLRLFIVRRPDDWKRFLDVYDPDRTEWPHIKAFMESDD

>SfurCSP4

MKCPLLSVSCLWISLLALSSSASAATKEKDPERKALYRLEYIDIEKVLDNNRMLTNFIRCFLRKGPCSPE

ARDFRKLLPKLAKTMCSDCSPRQRFIIKKVFKHLMEERPKEWELLMDRFDPQRKYAERLDTFMVDMTTPSTTTTTTSTTPSTPMSSTTQRIIEILRTSTEMSNESSP

>SfurCSP3

MQLLYALVFGCTLVMVSSDMPQSTYPTKYDDYNPDDILKNDRLFNQYFICLTKKKGCTTAGELLSAIIPD

ALATSCAKCSAKQKAIGEKVIRFLYFNKPDEFAEMSKIYDPEGKYLEMYIASGGLI

>SfurCSP2

MVLADTPTTSPKVETKAVESGKSSSKDEIPDQTFDRYINNERYMLMQYECLMGNKPCDHVGRKLKAAVPLVVRGLGCPKCSQREEDQMKRIVSHVQRSYPDKWQKLIKKYGN

>SfurCSP1

MFNLLTLVVCLSTIAVQIQAAPEEAQYTTKYDKINLDEILNNDRLFKSYFGCLMGGKCTPDGQTLRDILP

DALETACSKCSDTQKAGTEKVFKFMIEKKPSEFADLEKKYDPNGKYRARYEADAEKFGIKV

>NlugCSP11

MKSIILLVFVSMSAMVYRCRADEPSYPTSWDNVNIDEVLGNERLVQNYAKCLLEKGSCSPEGTELKKAIPDALKTGCTKCSDKQKAGAQKVIKWLVQKKPELWKEVVDKYDPSGEYTKKYEKEYQI

>NlugCSP10

MSEVLVTSLLFMLLACSELGSGQQQQNVDNIEMSIYDKMFENMDVNSLLKNHRLVDSYLKCFLNEGSCTHIGHEVKMMIPEVIKSRCGTCGENQMRALKAGLRLFIVLRPDDWQRFLDVYDPDRKEWPHIKAFMDSDD

>NlugCSP9

MKSQQLLVSCLFICTWLVVLMAPSANAAPKEKDPERKALYRLEYIDIEKVLDNNRMLTNFIRCFLRQGPCTPEARDFRKLLPKLAKTMCSDCTARQRYIIKKVFKHLMEERPKEWELLMDRFDPQRKYAERLDTFMVDMTTRAPVTSSPMPSSPVTLTSSSVTMSSTTQRVIEILRTSTDMSNESRPAS

>NlugCSP8

MSSTMLVFVAVLCFSAVLAKPADKYTTKYDNIDLDEVLSNQRLFDSYFKCLMGGKCTPDGQELRDALPDALATACEKCSEKQKEGTEKVMKFLIEKKPTEFAELEKKYDPQGTYRQKYKAEADKRGYSV

>NlugCSP7

MASASSGTTSTTSAPKTAESASAKSSSKDEIPDQTFDRYINNERYMLMQYECLMGNKPCDHVGRKLKAAVPLVVRGLGCPKCSQREEDQMKRIVSHVQRSYPDKWQKLIKKYGN

>NlugCSP6

MLWAARFIVLPLLFCVLQVWSAPADEKYTDIDFDSILANRRVLSSYVKCLTDKGPCTPQGKELKKIVPEVIQTSCTKCSPQQKKVVRNVITTMQSKYKDQWDLVVNKYDPKKQRSGELKAFLSGTD

>NlugCSP5

MRCLLLVAVVCAALVAVCHAQDSKYTSKYDNIDIDKILKNDRVLSQYIKCLMGEGSCTQEGRELKRLLPDAIQSNCSKCSEKQRSASVKVMRHLRQSRERDWNRLLDKYDPQGDKRKNLKLD

>NlugCSP4

MFLIAVWALSPRRLPWGLPWGGLAGVAAQQQAKNTRYTTRFDSIDVEVILKNERIFRRYMDCLLDKGRCTPEARELKRLLPEALKTECLKCSEVQRRQGAKVMAFIIKNKRPSWELLLAKYDPQGIFRAKYMYNENNIEAVLKQLEREQQGIYGTYSSTNSTTSSNSTSIR

>NlugCSP3

MKFLCVTIFECALIVVAFGMPQDTTYPTTYDDVNVDDILHNDRLFNRYFTCLTKKEGCTPEGKLLAATIP

DALATTCAKCSAKQKTAAEKVIKYLYFNKRDKFDELAKIYDPESNYLNKYLVDGFPAKV

>NlugCSP2

MSKLPVTLVLMLAVFSVDCGKLYKDRYTTKFDKIDLDEALNNQRLFESYLKCLMGDKCSPDGYELREALPDALATACAKCSEAQKAGTEKVIRFLIEKRPKEYALLEKKYDPEGIYRDKYKPIAEMKGIKLD

>NlugCSP1

MFKNVLLVCLLVAVVSAKPKPAEKKQYTTKYDNIDLDEILNNQRLFDNYYKCLLGGKCTPDGQELREALPDALATACSKCTEKQRVGTEKVIKYLIEKKPTEYSELEKKYDPQGNYKRKYQAEAAKRGIKV

>SaveCSP2

MDSRIAVVCVVLAVFAVDQTVGAPQKDALAAGSPTTYTNKYDHIDIDQVLASKRLVNSYVQCLLDKKPCTPEGAELRKILPDALKTQCAKCSATQKNAALKVVDRLQKDYDKEWKQLLDKWDPKREQFQKFQQFLTEEKKKGVVKF

>SaveCSP1

MAQLNLFVVLVASLVCFTLAEEKYSTKYENFDVDKVLNDDSLLTSYINCLLDEGNCTEEGQALKRVLPDALKTNCGKCTDTQKMKIEKILKFLMKNRSTDFDRLTAKYDPSGEYKKKLEKFSA

>MperCSP1

MNTLLLAVALCIAITMTVVQTAPAKYTTKYDNVNIDDILNNDRLVASYFKCLMETGKCTPEGEEIKRWLPEAIENKCENCSEKQKIGSEKIIKFLIEKKNDMWKQLEQKYDPQGLYKQRYSEEAKKLNLDV

>MperCSP2

MAHLNLFVVLVASLVCFTLAEEKYTTKFDNFDVDKVLNNNRILTSYIKCLLDEGNCTNEGRELRKVLPDALKTDCSKCTEVQKDRSEKVIKFLIKNRSTDFDRLTAKYDPSGEYKKKIEKFDSEKAAAAKH*

>MperCSP4

MTNNNMNSPRCRPEIFSLLAVAAIATVLVHQPSTVHCADAGVYPPQQQQQEATMFTAPSGYYVSTYDHMDVGRLLRNNKVVAGFVKCFTNEGPCTPEGRLAKAYLLPEIIRTVCGKCTPRQKDMARLVIRHIYTYRRGDFDKIMQIYDTDGKKNEIIDFMNQK

>MperCSP5

MNCKVLIALCCVAVYAAHASPAGAATAAAASADEEIKDFPAYMKRFDKLNVEQVLNNDRVLASHLKCFLNEGPCVQQSRDLKRVIPVIANNGCNGCTERQMTTIKKSLNFLRTKKPVEWARLVKIYDPSGTKLNKFLDA

>MperCSP6

MNTLLLAVALCIAITMTVVQTAPAKYTTKYDNVNIDDILNNDRLVASYFKCLMETGKCTPEGEEIKRWLPEAIENKCENCSEKQKIGSEKIIKFLIEKKNDMWKQLEQKYDPQGLYKQRYSEEAKKLNLDV*

>MperCSP7

MDRSSSSVTMKVFVIAVCVCAALARPEDSKVENKPAAVKSETLAAPLPTTIVKRATPQVVSTQQGASLPNVSEDVLDKALSDRRFVLRQLKCATGEGPCDPIGRKIKAHAPLVLRGMCVKCSQSEIKQIQRVMSHIQKNYPKEYTMMLKQYQSGF*

>MperCSP8

MTNNNMNSPRCRPEIFSLLAVAAIATVLVHQPSTVHCADAGVYPPQQQQQEATMFTAPSGYYVSTYDHMDVGRLLRNNKVVAGFVKCFTNEGPCTPEGRLAKAYLLPEIIRTVCGKCTPRQKDMARLVIRHIYTYRRGDFDKIMQIYDTDGKKNEIIDFMNQK*

>MperCSP9

MTSFCLNSVILMTITTVIVAHAASTGMTAFNNRSGSDIHMAQRDYNENKADKAEGFFFTITNFFSRRKHDDDKPDFITTFDIIRLLDEKYAMKQFYCVINKEPCDATGLRLKATIPEEINNDCERCTATETSNIRRILNYVKKHYPEFWDRVEPIYRNNMTA*

>MperCSP10

MVSKLFVSVFVLMSVVGVSYSVTEGDDDAAKVADKDLHPVNQEELKKFLSMMEKVDIDQILNNNRLMSNNVKCFLNEGPCTGQLREMKKMVPMLVKDSCSSCNKEQKNMMKKAMDAMKARRPNEYEQISKFFDPEGKYEKKFLENLNESK*

**Table S3** Primers used in qPCR.

| **Genes** | **Forward (5'-3')** | **Reverse (5'-3')** |
| --- | --- | --- |
| AlucOBP2 | AACCCTCAAGCGCTCCAGTT | CAATGGCGACCTGTGTTCTG |
| AlucOBP3 | CTTCGCTGTGGCCCTAACTC | CATTTGTCATCTTTGCCTGGTG |
| AlucOBP4 | GGCCACAATCCAAGGAGTTC | CAATTTGTTCGCTCACGCAG |
| AlucOBP7 | AACCGACAAGGCGAAGCA | AGAAATCTGGCGGACGAACTC |
| AlucOBP8 | AAGATGTTGGCACCAGAGCA | GCAACTCCTTATCCGTGTCGTAG |
| AlucOBP9 | ATGGAGAACCTGATAACA | GTATTCACACTTGCTCAT |
| AlucOBP11 | TTGGAAGGAAGTGATAGC | AGTCATACAGTTGAAGGAA |
| AlucOBP15 | AAGAAGACTGTCATGCTGGATGT | CATTCACTCTTGCCTTTGGTATCTC |
| AlucOBP16 | TCGGCTACTTGACAGGTGGAC | CTTTCTTCCCGTTTGTAACACTCTG |
| AlucOBP17 | GAGAGAAATGGCTCAGGGACTT | CGAAATGACTCCGAGGTTGC |
| AlucOBP18 | CTCCGCCCTTCTTGTGGCTTAT | GCAAGAAGCACCTTTCAGCAT |
| AlucOBP19 | GCCAAAATCGCAGAAATCAAA | GTCAGCGGGGTCCTCGTAT |
| AlucOBP22 | TCAAGGACCACCCTGAGAAAC | GCCAAGTCGCATTCGTTGT |
| AlucOBP23 | TTCACTGTAGCCTTGTCTT | CTTCTTCAAAGCACTTATGGA |
| AlucOBP26 | AAAAGTCAAAGTTCAGTGCCAGC | ATCCCGCCAGTCTTCGTGT |
| AlucOBP27 | CCTCGTCGCTTTGTTGGTATT | CTGTCGAATTTTTACACTGTTTTAGA |
| AlucOBP28 | AGCGAAGAAAGTGATGACCAAA | TCGGGAATGTAGTCTAAAAACGG |
| AlucOBP29 | GAGACCACCTCAATGTTGCGAT | CCTGTTGCCGATTTGCCTG |
| AlucOBP31 | TTATGGACTGAGAGCACGGACT | GATGATGACCGATTGGCACCT |
| AlucOBP35 | GAGCCCGAATGATAAAGAACGA | TTCAGACGGGTTCTCACCATTAA |
| AlucCSP2 | TATAGGAGCGGTCGGTAT | CTGGTACAACCTTTGATTCC |
| AlucCSP3 | GCTCTATCGGCTGAAGTT | GGTGAGGATTCGGTCATT |
| AlucCSP4 | GAATGCCTCACGAACAAG | CGGAACACTTGGAACAAC |
| AlucCSP9 | GACCAACTGTTCCAAGTG | CATCTTCGTACCTCTTCCT |
| AlucCSP10 | GTAGCGTTTGTCCTTCAG | GCATAGCTGCCTGTTTAG |
| AlucCSP12 | GACAAGCAGAAGGGGATA | TCGTAGATGGACAGGATTC |
| AlucCSP16 | GACAAGCAGAAGGGGATA | TCGTAGATGGACAGGATTC |
| AlucCSP17 | GACAGCATCCTCATCAATC | CGCAATTCTGAGATCCTTC |
| AlucOR109 | CGTGGTTGAGTCTCTTCT | CATCGTAGTTGCTGGATTC |
| AlucIR21a | GCCTCTTCTTGTGAGTATTG | CTCTTCCCATTCCAGTTCT |
| AlucSNMP2a | GCCATCGTCTTCCTCATA | GAGTCTTCTTCCAGCACTA |
| GAPDH | CGAGTTCCTGTCCCTAATGTTTC | GCCTCCTTCACCTTCTGCTT |

**Table S4** Statistical results of data quality in female tissue samples.

| Sample | Female forelegs | Female middle legs | Female hind legs |
| --- | --- | --- | --- |
| Raw Reads Number | 106,020,038 | 96,631,626 | 110,435,520 |
| Raw Bases Number | 15,903,005,700 | 14,494,743,900 | 16,565,328,000 |
| Clean Reads Number | 102,528,364 | 93,578,672 | 106,681,660 |
| Clean Bases Number | 15,379,254,600 | 14,036,800,800 | 16,002,249,000 |
| Clean Reads Rate(%) | 96.71 | 96.84 | 96.6 |
| Raw Q30 Bases Rate(%) | 94.67 | 94.75 | 94.27 |
| Clean Q30 Bases Rate(%) | 95.01 | 95.05 | 94.65 |

**Table S5** Statistical results of data quality in male tissue samples.

| Sample | Male forelegs | Male middle legs | Male hind legs |
| --- | --- | --- | --- |
| Raw Reads Number | 104,039,236 | 90,768,110 | 106,745,344 |
| Raw Bases Number | 15,605,885,400 | 13,615,216,500 | 16,011,801,600 |
| Clean Reads Number | 100,468,622 | 87,850,168 | 103,088,688 |
| Clean Bases Number | 15,070,293,300 | 13,177,525,200 | 15,463,303,200 |
| Clean Reads Rate(%) | 96.57 | 96.78 | 96.57 |
| Raw Q30 Bases Rate(%) | 94.56 | 94.53 | 94.59 |
| Clean Q30 Bases Rate(%) | 94.93 | 94.85 | 94.92 |

| DEG Set | All DEG | up-regulated | down-regulated |
| --- | --- | --- | --- |
| Female forelegs vs Female middle legs | 358 | 190 | 168 |
| Male middle legs vs Female middle legs | 418 | 266 | 152 |
| Male middle legs vs Male forelegs | 562 | 348 | 214 |
| Female forelegs vs Male forelegs | 669 | 331 | 338 |
| Female hind legs vs Female middle legs | 1347 | 849 | 498 |
| Female hind legs vs Female forelegs | 1812 | 1086 | 726 |
| Female hind legs vs Male hind legs | 2186 | 868 | 1318 |
| Male middle legs vs Male hind legs | 3141 | 1776 | 1365 |

| Male hind legs vs Male forelegs | 3519 | 1989 | 1530 |
| --- | --- | --- | --- |

|  |  |  |  |
| --- | --- | --- | --- |

**Table S6** The number of differentially expressed genes in different DEG Sets.

| Gene name | FPKM | | | | | |
| --- | --- | --- | --- | --- | --- | --- |
|  | Male | | | Female | | |
|  | Forelegs | Middle legs | Hind legs | Forelegs | Middle legs | Hind legs |
| OBP2 | 17.87 | 13.72 | 2.15 | 15.51 | 11.10 | 5.48 |
| OBP3 | 821.73 | 669.62 | 30.43 | 560.70 | 383.32 | 98.70 |
| OBP4 | 5.29 | 3.79 | 1.98 | 5.38 | 2.94 | 3.79 |
| OBP7 | 98.43 | 71.14 | 6.36 | 95.98 | 59.12 | 24.20 |
| OBP8 | 6.62 | 7.09 | 0.57 | 2.16 | 1.24 | 1.18 |
| OBP9 | 8293.34 | 5729.10 | 362.41 | 5427.16 | 3725.80 | 1267.98 |
| OBP11 | 14.30 | 9.56 | 1.18 | 10.34 | 7.70 | 2.89 |
| OBP15 | 189.54 | 226.31 | 45.17 | 169.98 | 205.37 | 105.91 |
| OBP16 | 5.77 | 5.75 | 1.00 | 3.88 | 4.94 | 2.08 |
| OBP17 | 6.46 | 7.37 | 0.38 | 1.40 | 1.07 | 1.36 |
| OBP18 | 14.47 | 20.53 | 12.97 | 17.45 | 19.78 | 7.47 |
| OBP19 | 845.26 | 606.54 | 25.48 | 647.59 | 438.78 | 137.51 |
| OBP22 | 38.08 | 14.72 | 2.73 | 7.42 | 4.96 | 3.70 |
| OBP23 | 262.15 | 190.11 | 24.34 | 191.30 | 143.23 | 72.09 |
| OBP26 | 343.12 | 323.91 | 126.64 | 277.49 | 258.46 | 213.09 |
| OBP27 | 91.60 | 76.41 | 16.29 | 69.18 | 48.20 | 25.82 |
| OBP28 | 14.05 | 12.63 | 2.73 | 11.09 | 9.51 | 6.00 |
| OBP29 | 65.24 | 69.36 | 25.44 | 51.89 | 54.42 | 50.23 |
| OBP31 | 23.89 | 16.92 | 1.81 | 16.65 | 11.11 | 3.62 |
| OBP35 | 65.41 | 46.91 | 2.25 | 51.60 | 33.86 | 11.83 |
| CSP2 | 1442.57 | 1699.05 | 601.82 | 1244.36 | 1318.17 | 1017.00 |
| CSP3 | 244.65 | 124.07 | 24.48 | 128.48 | 73.98 | 38.69 |
| CSP4 | 392.32 | 340.09 | 103.82 | 264.28 | 235.54 | 132.81 |
| CSP9 | 2888.48 | 2584.97 | 702.40 | 1960.07 | 1808.07 | 1008.74 |
| CSP10 | 21.34 | 14.93 | 4.08 | 13.98 | 9.37 | 6.87 |
| CSP12 | 12.80 | 11.05 | 1.42 | 4.99 | 3.84 | 2.72 |
| CSP16 | 13.29 | 4.57 | 0.84 | 1.79 | 2.00 | 0.91 |
| CSP17 | 7.01 | 7.95 | 3.13 | 6.48 | 6.91 | 5.61 |
| OR109 | 6.48 | 6.32 | 2.91 | 6.12 | 5.94 | 7.67 |
| IR21a | 78.83 | 95.62 | 109.26 | 73.57 | 84.94 | 137.37 |
| SNMP2a | 6.60 | 5.94 | 1.91 | 3.34 | 3.77 | 2.58 |

**Table S7** The FPKM values of chemosensory genes in male- and female- legs transcriptome of *A*. *lucorum*.
